# Evans blue-modified radiolabeled fibroblast activation protein inhibitor as long-acting cancer therapeutics

**DOI:** 10.7150/thno.68182

**Published:** 2022-01-01

**Authors:** Xuejun Wen, Pengfei Xu, Mengqi Shi, Jia Liu, Xinying Zeng, Yiren Zhang, Changrong Shi, Jingchao Li, Zhide Guo, Xianzhong Zhang, Pek-Lan Khong, Xiaoyuan Chen

**Affiliations:** 1 State Key Laboratory of Molecular Vaccinology and Molecular Diagnostics & Center for Molecular Imaging and Translational Medicine, School of Public Health, Xiamen University, 4221-116 Xiang'An South Rd, Xiamen 361102, China;; 2 Institute of Clinical Pharmacy & Pharmacology, Jining First People's Hospital, Jining Medical University, Jining 272000, China;; 3 Department of Diagnostic Radiology, Yong Loo Lin School of Medicine, National University of Singapore, 119074, Singapore;; 4 Nanomedicine Translational Research Program, NUS Center for Nanomedicine, Yong Loo Lin School of Medicine, National University of Singapore, Singapore 117597, Singapore; 5 Clinical Imaging Research Centre, Centre for Translational Medicine, Yong Loo Lin School of Medicine, National University of Singapore, Singapore 117599, Singapore; 6 Departments of Surgery, Chemical and Biomolecular Engineering, and Biomedical Engineering, Yong Loo Lin School of Medicine and Faculty of Engineering, National University of Singapore, Singapore, 119074, Singapore

**Keywords:** fibroblast activation protein (FAP), albumin binder, FAPI, SPECT imaging, radioligand therapy

## Abstract

**Rationale:** Fibroblast activation protein (FAP) targeted molecular imaging radiotracers have shown promising preclinical and clinical results in tumor diagnosis. However, rapid clearance and inadequate tumor retention of these molecules have hindered them for further clinical translation in cancer therapy. In this study, we aimed to develop a series of albumin binder-truncated Evans blue (EB) modified FAP targeted radiotracers, and optimize the pharmacokinetic (PK) characteristics to overcome the existing limitations in order to apply in the radionuclide therapy of cancer.

**Methods:** A series of compounds with the general structure of EB-FAPI-Bn were synthesized based on a FAP inhibitor (FAPI) variant (FAPI-02) and radiolabeled with ^177^LuCl_3_. To verify the binding affinity and FAP targeting specificity of these tracers *in vitro*, U87MG cell uptake and competition assays were performed. Preclinical PK was evaluated in U87MG tumor-bearing mice using SPECT imaging and biodistribution studies. The lead compound EB-FAPI-B1 was selected and cancer therapeutic efficacy of ^177^Lu-EB-FAPI-B1 was assessed in U87MG tumor-bearing mice.

**Results:** ^177^Lu-EB-FAPI-B1, B2, B3, B4 were stable in PBS (pH 7.4) and saline for at least 24 h. EB-FAPI-B1 showed high binding affinity (IC_50_ = 16.5 nM) to FAP *in vitro*, which was comparable with that of FAPI-02 (IC_50_ = 10.9 nM). SPECT imaging and biodistribution studies of ^177^Lu-EB-FAPI-B1, B2, B3, B4 have proved their prominently improved tumor accumulation and retention at 96 h post-injection, especially for ^177^Lu-EB-FAPI-B1, high tumor uptake and low background signal make it the optimal compound. Compared to the saline group, noteworthy tumor growth inhibitions of ^177^Lu-EB-FAPI-B1 have been observed after administration of different dosages.

**Conclusion:** In this study, several EB modified FAPI-02 related radiopharmaceuticals have been synthesized successfully and evaluated. High binding affinity and FAP targeting specificity were identified *in vitro* and *in vivo*. Remarkably enhanced tumor uptake and retention of EB-FAPI-B1 were found over the unmodified FAPI-02. ^177^Lu-EB-FAPI-B1 showed remarkable tumor growth suppression in U87MG tumor model with negligible side effects, indicating that ^177^Lu-EB-FAPI-B1 is promising for clinical application and transformation.

## Introduction

Targeting neoplastic cells has been used as a conventional method for tumor growth and spread determination, however, with the key function of cancer associate fibroblasts (CAFs) being recognized, scientists have been striving to target CAFs for diagnosis and antitumor therapy. CAFs constitute a predominant portion of non-malignant tumor stroma, which in turn account for 90% of the mass in tumors with desmoplastic reactions; and play an essential role in tumor growth, migration and progression 1, 2. A distinguishing characteristic of CAFs is overexpression of the serine protease fibroblast activation protein (FAP), the presence of which confers a poor prognosis and fast progression of disease in cancer patients 3, 4. As a result, targeting FAP for both imaging and delivery of the therapeutics has become an attractive approach 5.

To target FAP for imaging, early attempts were made to develop radiolabeled antibodies. Iodine 131-labeled FAP specific monoclonal antibody F19 (^131^I-mAbF19) was evaluated in patients with hepatic metastases from colorectal carcinoma. However, the long circulation time of intact antibodies hampered the delivery of radioactive agents, and suggested that smaller molecules may be suitable for radionuclide imaging 6, 7. In recent years, the development of a quinoline-based small-molecule radiopharmaceutical based on a FAP-specific inhibitor (FAPI) has revolutionized the strategy for FAP-targeted theranostics. These synthesized FAPIs were able to show specific uptake, rapid internalization, and successful imaging of tumors *in vivo* and in clinical studies. Nevertheless, due to the enzymatic deiodination with efflux of free iodine of FAPI-01, prolonged incubation period results in lower intracellular radioactivity. To solve this issue, FAPI-02 was synthesized to have the FAP-targeting group chemically linked to the chelator DOTA, resulting in a theranostic compound with favorable pharmacokinetic and biochemical properties 2. Indeed FAPI-02 acts as an ideal tracer that specifically bind to its target protein FAP to ensure reliable distinction between cancerous and normal tissue as well as achieve a low background signal, resulting in high image contrast. However, with regards to potential tracer's applications for therapeutic purposes, improvement needs to be made to allow: 1) even longer circulation half-life that increases tumor uptakes; 2) slower efflux/longer tumor retention; 3) higher tumor to normal tissue contrast 8. This stimulated further modifications to the lead structure FAPI-02 to achieve enhanced tumor retention and high target specificity 9-11. Although FAPI-04, -21, -46 and other similar structures were developed afterwards and outperformed FAPI-02 in achieving prolonged tumor retention while retaining, if not declining, uptake by normal tissues 12-14. Subtle structural modification to the lead structure itself certainly is a practical and promising strategy to improve pharmacokinetics (PK) and pharmacodynamics (PD), yet it remains challenging to achieve massive improvement in PK and PD properties.

With this in mind, we aim to improve PK and PD profiles of the lead structure FAPI-02, by adding functional chemical groups without significantly affecting the desired biological activity, in order to accomplish a more substantial enhancement in tumor retention while keeping the normal tissue uptake at low level. Albumin serves as a versatile carrier for drug delivery 15, 16. As the association of bioactive drug to albumin is a reversible action, the albumin-drug complex functions as a drug reservoir that can enhance the drug distribution and bioavailability 17. Evans blue (EB) is a good example of albumin binding moiety that exhibits relatively high affinity for binding site 1 on serum albumin. Previous studies showed that conjugation of EB derivatives onto targeting molecules remarkably prolongs the blood circulation of drugs, thus improves the therapeutic effectiveness 18, 19, such as ^177^Lu-EB-PSMA for the therapy of castration-resistant prostate cancer 20, 21; ^177^Lu-EB-TATE for the treatment of neuroendocrine tumors 22; ^90^Y-EB-RGD for the treatment of glioblastoma 23. Inspired by this improved radiotherapeutic efficacy, this research focuses on investigating whether EB conjugation of FAPI-02 improves the efficacy of FAP-targeting radioligand therapy, especially in regards to tumor retention, tumor-to-normal tissue contrast as well as treatment response.

Glioblastoma multiforme (GBM) is the most aggressive glioma of WHO Grade III and IV. Despite the advances in therapeutics, the prognosis for patients remains poor 24, 25. FAP was found to be expressed on glioma cells and tumor stroma especially in proximity to blood vessels 26. Therefore, several studies have been performed to evaluate the PK and biodistribution of ^68^Ga-FAPI-02/04 in glioblastoma patients and achieved the positive results 27, 28. But up to now, the therapeutic effects of FAPI-02/04 in glioblastoma models have not been researched.

In this study, EB-FAPI-B1, B2, B3 and B4 were designed and synthesized with several major components: a quinoline-based FAPI-02 as lead structure; a 1,4,7,10-tetraazacyclododecane-1,4,7,10-tetraacetic acid (DOTA) group that enables radionuclide labeling; poly(ethylene glycol) (PEG, with or without); and truncated EB moiety as an albumin binder. U87MG tumor-bearing mice were selected as the animal model. The stability and specificity of these radiotracers were firstly confirmed before pursuing a series of investigation and comparison of PK and PD profiles utilizing small-animal SPECT and PET imaging, biodistribution and radiotherapy analysis. The results demonstrated a strong affinity and specificity of ^177^Lu-EB-FAPI-B1 to U87MG tumor cells, an enhanced tumor retention and tumor-to-background contrast, as well as a great potency in radiotherapy of U87MG tumor-bearing mice.

## Materials and Methods

### General

The glioblastoma cell line, U87MG, was purchased from the China National Infrastructure of Cell Line Resource. ^177^LuCl_3_ in a solution of 0.05 M hydrochloride was purchased from ITG (Germany). Radioactivity was measured using a CRC-25R Dose Calibrator (CAPIN-TEC Inc., USA) and γ-counter (WIZARD 2480; Perkin-Elmer, USA). Radiolabeling efficiency and radiochemical purity were tested using a Mini-Scan Instant Thin Layer Chromatography Scanner (TLC; BioScan, USA) and Dionex Ulti-Mate 3000 high performance liquid chromatography (HPLC; Thermo Scientific, USA), with a Raytest radioactivity detector (BioScan, USA). SPECT imaging was performed using a nanoScan SPECT/CT scanner (Mediso, Hungary).

### Chemistry and Radiochemistry

**Figure 1:**
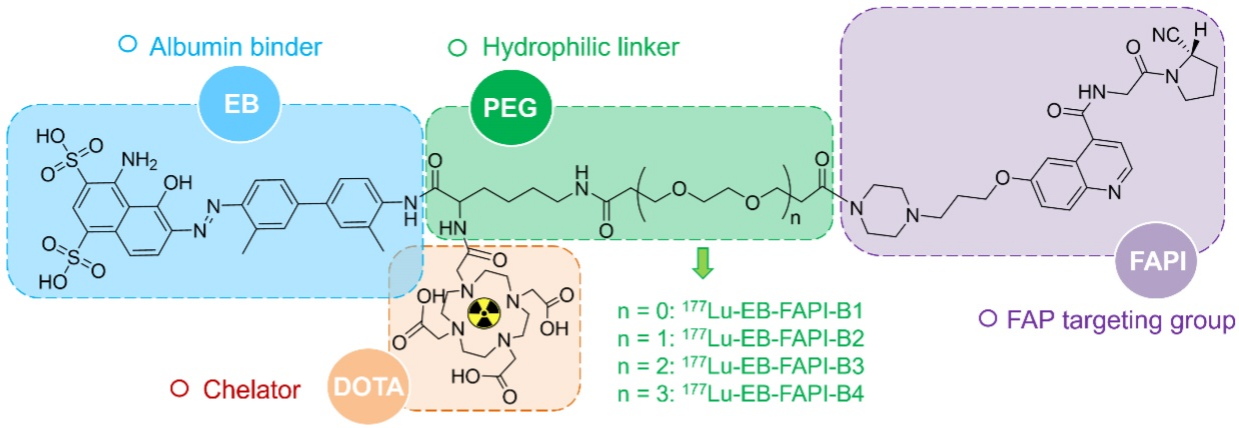
Chemical structure and each part of the functional groups of ^177^Lu-EB-FAPI-B1 (without PEG), ^177^Lu-EB-FAPI-B2 (with PEG: n = 1), ^177^Lu-EB-FAPI-B3 (with PEG: n = 2) and ^177^Lu-EB-FAPI-B4 (with PEG: n = 3).

A detailed scheme of chemical synthesis protocol is elaborated in the supplementary materials. To radiolabel the precursor with ^177^Lu, 185-740 MBq of ^177^LuCl_3_ was firstly diluted with 0.2 mL of 0.5 M NH_4_OAc (pH = 5.5) before incubating with EB-FAPI-Bn (50 μg dissolved in 10 μL dimethyl sulfoxide). The mixture was heated and thoroughly mixed for 30 min at 95 °C. Radiolabeling efficiency and radiochemical purity were tested using thin-layer chromatography (TLC) and high-performance liquid chromatography (HPLC). Radiolabeling of ^68^Ga was performed by incubating with 50 μg precursor (pH = 4.5-5.0) at 95 ℃ for 10 min. The ^68^Ga-labeled product was purified by C18 column extraction and the radiochemical purity was determined by HPLC. Stability of ^177^Lu-EB-FAPI-B1, ^177^Lu-EB-FAPI-B2, ^177^Lu-EB-FAPI-B3 and ^177^Lu-EB-FAPI-B4 (See **Figure 1** for the structures) in PBS and saline were monitored up to 24 h using radio-HPLC. More information about radiochemistry, quality control and octanol/water partition coefficient can be found in supplemental information.

### Cell Uptake and Binding Assays

U87MG cells were cultivated in Dulbecco modified Eagle medium (DMEM) containing 10% fetal calf serum (FCS) at 37 ℃ in 5% carbon dioxide (CO_2_). For cell uptake experiment, U87MG cells were seeded in 24-well plates (2 × 10^5^/well) overnight. The cells were incubated with 37 kBq of ^177^Lu-EB-FAPI series compounds in 0.5 mL of medium for 0.5, 1, 2, 4, 8 and 24 h at 37 °C. In the blocking group, FAPI-02 (10 μg/well) were added as inhibitors of the labeled precursors. For competition assays, U87MG cells were incubated simultaneously with different concentrations (10^-5^-10^-11^ M) of unlabeled FAPI-02 or EB-FAPI-B1 and ^68^Ga-FAPI-02 in 0.4 mL of fresh medium for 1 h. At the corresponding time points, the medium was removed and the cells were washed twice with cold PBS (pH 7.4) and subsequently lysed with 1 mL of NaOH (0.1 M). Cell lysates was collected and the radioactivity was determined using a γ-counter. The cell uptake experiment was repeated four times for each time point and the competition assays were repeated three times at different concentrations.

### Molecular Docking Modeling Method

To learn about the visualized interaction between FAPI-02 or EB-FAPI-B1 and proteins, including FAP and HSA, molecular docking technique was performed in MOE v2018.0101 29. The 3D structures of FAP and albumin were downloaded from RCSB Protein Data Bank with PDB ID of 1Z68 and 5YOQ, respectively. Prior to docking, the force field of AMBER10:EHT and the implicit solvation model of Reaction Field (R-field) were selected. MOE-Dock was used for molecular docking simulations of the proteins with compounds.

### U87MG Tumor Model

All animal studies were approved by the Animal Care and Use Committee of Xiamen University and carried out in compliance with the national laws related to the conduct of animal experimentation. 6-week-old female BALB/c nude mice from Beijing Vital River Laboratory Animal Technology Co., Ltd. were used in this study. The right upper limbs of BALB/c nude mice were subcutaneously inoculated with the suspension of U87MG cells (5 × 10^6^ cells in 100 μL of PBS). The mice underwent radioligand therapy experiments when the tumor volume reached about 100 mm^3^ and small animal SPECT imaging studies when the tumor volume reached about 500 mm^3^.

### Small Animal SPECT and PET Imaging

SPECT/CT imaging studies were performed using a nanoSPECT/CT preclinical scanner (Mediso, Hungary). About 37 MBq of radiotracers were injected intravenously into female ICR mice and U87MG tumor-bearing mice, whole body SPECT images were acquired at 1, 4, 24, 48, 72 and 96 h p.i. The acquisition parameters were as follows: 56.1, 112.9 and 208.4 keV energy peaks for ^177^Lu, window width of 20%, matrix of 256 × 256, medium zoom, and 48 frames. Mice were anaesthetized using 1.5% isoflurane to maintain spontaneous breathing during imaging.

PET scans were performed by using Inveon small-animal PET scanner (Siemens Preclinical Solution). For the 10-min static PET imaging, about 7.4 MBq of ^68^Ga-FAPI-02 or ^68^Ga-EB-FAPI-B1 were given to U87MG tumor bearing mice through tail-vein injection. At 1 h p.i., the mice were anaesthetized and placed on the bed for PET imaging. The block assay was performed by injection of EB-FAPI-B1 to the U87MG tumor mice before the administration of ^68^Ga-FAPI-02. PET images were reconstructed with attenuation correction and analyzed through drawing regions of interest (ROIs).

### Biodistribution Study

In biodistribution study of normal mice, healthy ICR mice (18-20 g) were randomly divided into 6 groups (n = 4/group), injected with radiotracers (740 kBq in 0.1 mL saline) and sacrificed at 0.25, 1, 4, 8, 24 and 48 h p.i. Tissues and organs of interest were collected, wet weighed, and counted by a γ-counter. In addition, BALB/c nude mice bearing U87MG tumors were injected with radiotracers for SPECT imaging, and then, sacrificed at 96 h p.i., the major organs and tumors were collected and weighed. Radioactivity was detected using a γ-counter, and the results were calculated as percentage of injected dose per gram (%ID/g).

### Radiotherapy Study and Histopathological Analysis

Two weeks after inoculation of U87MG tumor cells, the average tumor volume reached about 100 mm^3^ in BALB/c nude mice. The U87MG tumor-bearing mice were randomly divided into 4 groups (n = 8/group), and subsequently treated with saline, 30 MBq ^177^Lu-EB-FAPI-B1, 18.5 MBq ^177^Lu-EB-FAPI-B1, 7.4 MBq ^177^Lu-EB-FAPI-B1, respectively. Tumor volumes and body weights of all mice were monitored every two days. Individual tumor size was calculated with the formula: length × width^2^/2. The mice were euthanized when the tumor volume exceeded 1500 mm^3^. Histopathologic staining was performed with an anti-human FAP mAb (ab207178, Abcam). Hematoxylin and eosin (H&E) staining and terminal deoxynucleotidyl transferase-mediated dUTP-biotin nick-end labeling (TUNEL) analysis were carried out using a commercially available kit (Beyotime).

### Statistical Analysis

Quantitative data were expressed as mean ± SD. All statistical analyses were conducted using GraphPad Prism 7.0 statistical software. One-way ANOVA of variance was used to analyze the differences between the control and the experimental groups. A p-value < 0.05 was considered statistically significant.

## Results

### Chemistry and Radiochemistry

**Figure 2:**
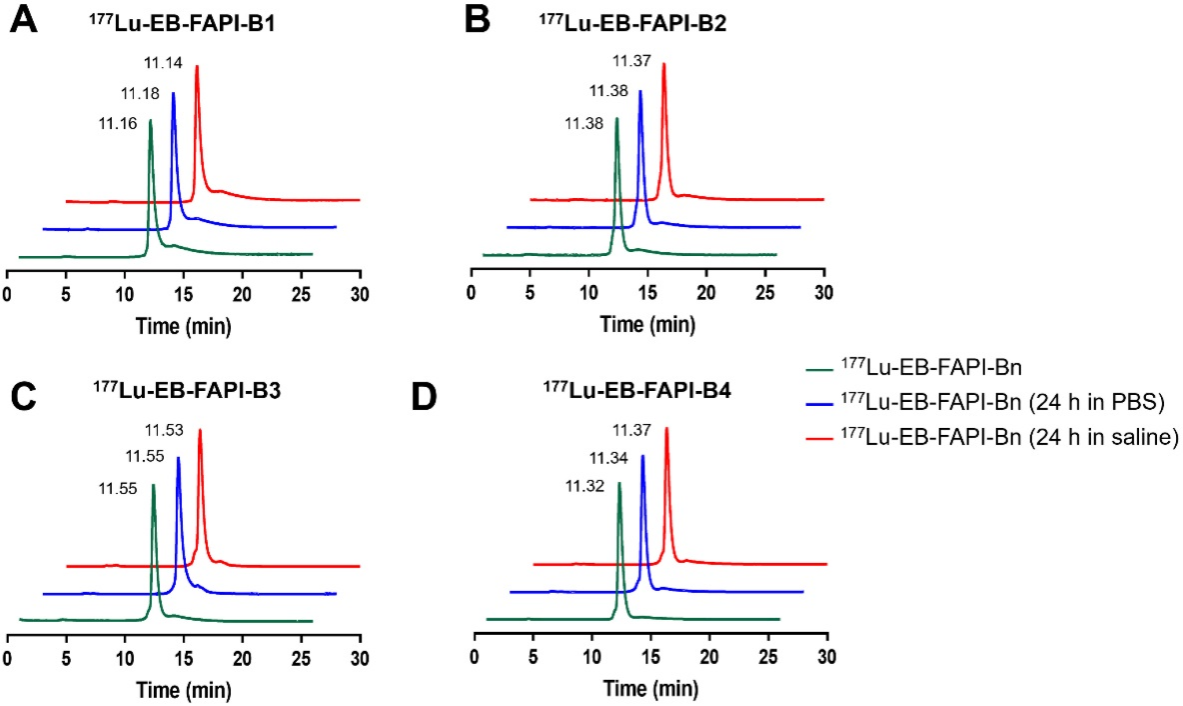
*In vitro* stability analysis of ^177^Lu-EB-FAPI-B1, B2, B3 and B4 after incubation in PBS (pH 7.4) and saline for 24 h.

Among all the synthesized EB-FAPI-Bn, EB-FAPI-B1 and its intermediate products were characterized by MALDI TOF-MS, ^1^H NMR, ^13^C NMR and HPLC (**Figure S1-S14**), with their structural formulas shown in **Figure 1**. As shown in **Table S1**, the radiolabeling yield and the radiochemical purity of these radiotracers were over 97%, the specific activity of ^177^Lu-EB-FAPI-B1, ^177^Lu-EB-FAPI-B2, ^177^Lu-EB-FAPI-B3 and ^177^Lu-EB-FAPI-B4 were 5.87-11.7, 6.2-12.4, 6.55-13.1, 6.85-13.7 MBq/nmol, respectively. Octanol/water partition coefficients (expressed as Log P) of the four radiotracers were -1.90 ± 0.12, -2.10 ± 0.04, -2.13 ± 0.11 and -2.18 ± 0.004, respectively, indicating a trend in increased hydrophilicity along with PEG prolongation. HPLC and TLC analysis results showed that ^177^Lu-EB-FAPI-B1, ^177^Lu-EB-FAPI-B2, ^177^Lu-EB-FAPI-B3 and ^177^Lu-EB-FAPI-B4 have high stability in PBS and saline, the radiochemistry purities were both still over 95% even at 24 h and 48 h (**Figure 2 and Figure S15**).

### High Cellular Uptake and Specificity to FAP

**Figure 3:**
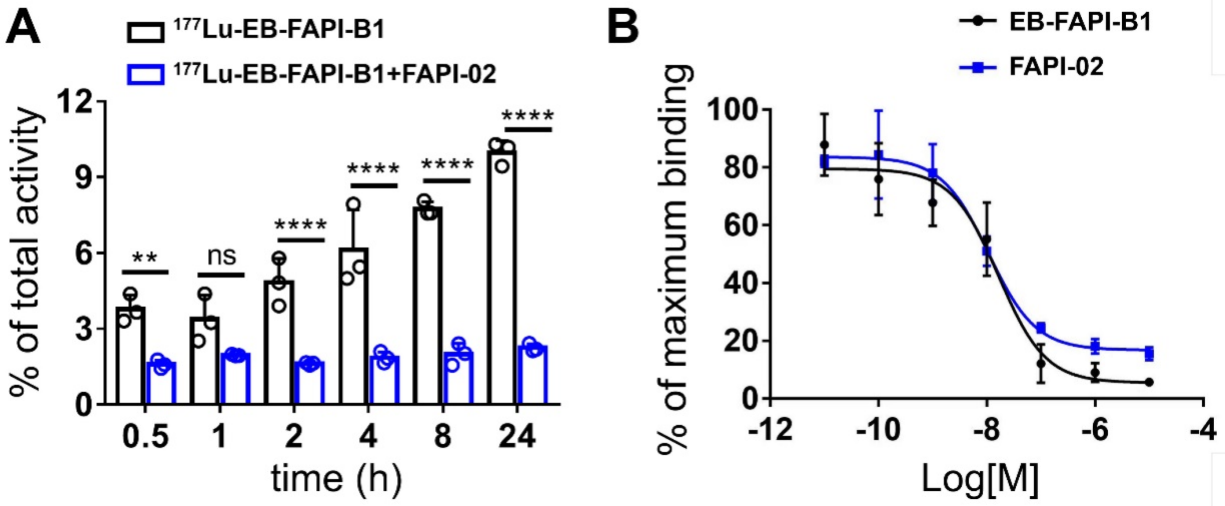
** (A)** Cellular uptake assay of ^177^Lu-EB-FAPI-B1 and blocking experiment by FAPI-02 in U87MG tumor cells. **(B)** Competition assays of EB-FAPI-B1 (IC_50_: 1.65 × 10^-8^ M) and FAPI-02 (IC_50_: 1.09 × 10^-8^ M).

In FAP-positive U87MG cells, the series of radiotracers have a gradually increased uptake over time until 24 h. The highest cellular uptake of ^177^Lu-EB-FAPI-B1 was observed after incubation for 24 h (up to 9.97 ± 0.42%). Cell uptake of the radiolabeled tracers could be blocked by the addition of unlabeled EB-FAPI-B1 (decreased to 2.25 ± 0.14%), proving the specific binding between ^177^Lu-EB-FAPI-Bn and FAP (**Figure 3A, Figure S16**). The ligand concentrations required for 50% inhibition (IC_50_) of EB-FAPI-B1 and FAPI-02 were 1.65 × 10^-8^ M and 1.09 × 10^-8^ M, respectively (**Figure 3B**). The comparable IC_50_ values of EB-FAPI-B1 and FAPI-02 suggest that the modification with the truncated EB structure has minimal influence in FAP binding affinity.

### Molecular Docking Modeling Studies

**Figure 4:**
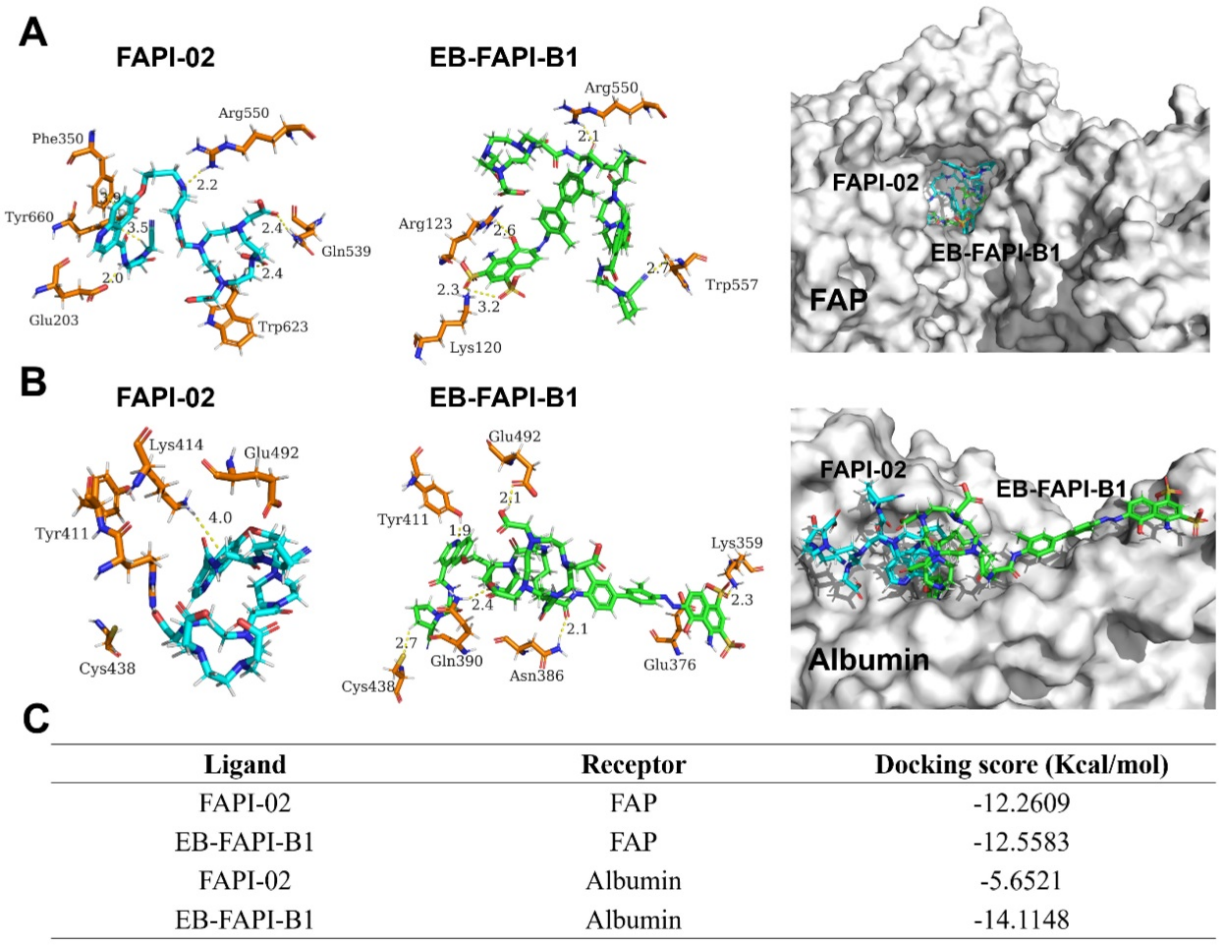
** (A)** Docking of FAPI-02 and EB-FAPI-B1 to FAP; **(B)** Docking of FAPI-02 and EB-FAPI-B1 to albumin. FAPI-02 is colored in cyan, EB-FAPI-B1 is colored in green, the surrounding residues in the binding pocket are colored in orange. The backbones of the receptors are shown in white surface and the hydrogen bonds are yellow dashed lines. **(C)** Docking scores of ligands-receptors for FAPI-02 and EB-FAPI-B1.

The docking modeling results of FAPI-02 and EB-FAPI-B1 with FAP are depicted in **Figure 4A**. Both FAPI-02 and EB-FAPI-B1 have formed an appropriate steric complementarity with the binding site of FAP. Within the binding pocket, little difference was observed by comparing the amino acid residues and non-covalent bonding between docking of EB-FAPI-B1 and FAPI-02 to FAP. The docking scores of EB-FAPI-B1 and FAPI-02 to FAP exhibited that EB-FAPI-B1 provided similar free energy of binding to FAP than FAPI-02, at -12.56 kcal/mol and -12.26 kcal/mol, respectively (**Figure 4C**). Collectively, FAPI-based structure modified with EB moiety did not down-tune its affinity and specificity towards FAP.

Another goal of this research is to determine the albumin binding affinity of the functional molecules. As shown in **Figure 4B**, according to the modeling results of HSA to EB-FAPI-B1, multiple amino acid residues and non-covalent bonding were observed between EB-FAPI-B1 and HSA, and the primary regions of intermolecular binding situated in hydrophobic cavities. But for FAPI-02 with HSA, only the nitrogen atom of Lys^414^ with the benzene ring of FAPI-02 formed H-π interaction was observed. The docking score of protein-ligand interaction was calculated (**Figure 4C**), and the binding energies of EB-FAPI-B1 and FAPI-02 to HSA were -14.11 kcal/mol and -5.65 kcal/mol, respectively, which demonstrated that EB-FAPI-B1 and HSA has significantly higher binding free energy than FAPI-02.

### Pharmacokinetic Characterization of Radiotracers *in vivo*

To evaluate the pharmacokinetic characterization of these radiotracers *in vivo*, SPECT imaging was conducted in healthy ICR mice for different times. As shown in **Fig. S17**, ^177^Lu-EB-FAPI-B1, B2, B3, and B4 demonstrated a significant uptake in the blood at 1 h and 4 h post-injection (p.i). The radioactive signal declined in the heart and increased in the kidneys over time. At 24 h and 48 h p.i., the radioactivity in the heart was barely observed while the kidneys possessed a sustained radioactivity. Characteristically, ^177^Lu-EB-FAPI-B1 showed low background signals in most organs except for the kidneys, while signal in the kidneys declined at 48 h as compared with that at 24 h p.i. However, higher radioactivities of ^177^Lu-EB-FAPI-B2, B3, B4 retained in the liver and kidneys until 48 h p.i. The continuously high radioactivity in the liver and kidneys would result in toxicity to normal organs, which make the three radiotracers less suitable for radioligand therapy *in vivo*.

**Figure 5:**
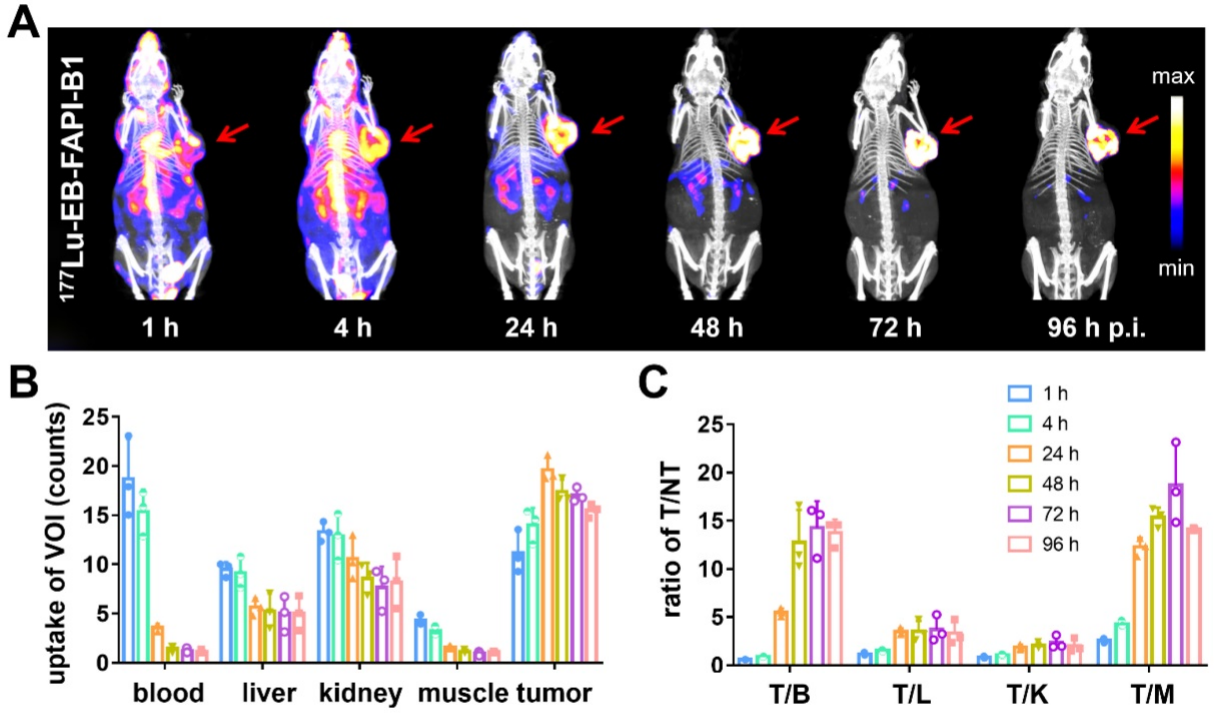
** (A)** SPECT/CT imaging of ^177^Lu-EB-FAPI-B1 in U87MG tumor bearing mice at 1, 4, 24, 48, 72 and 96 h post injection (p.i.);** (B)** The uptake of ^177^Lu-EB-FAPI-B1 in the blood, liver, kidneys, muscle and tumor at different time points p.i.; **(C)** The ratios of target/nontarget organs at different time points of SPECT imaging.

Representative whole-body SPECT images of U87MG tumor-bearing mice are shown in **Figure 5A** and **Figure S18A**. At 1 h after injection of ^177^Lu-EB-FAPI-B1, B2, B3, B4, most of radioactivity accumulated in the heart and blood vessels, and the background signal was also high. At 4 h p.i., obvious tumor uptake was observed for ^177^Lu-EB-FAPI-B1, B2, but not for ^177^Lu-EB-FAPI-B3, B4. As shown in **Figure 5B-C**, in the case of ^177^Lu-EB-FAPI-B1, high tumor uptake and tumor-to-muscle ratio (T/M: 12.29 ± 0.85) were observed at 24 h p.i., with the peak T/M value occurred at 72 h p.i. (18.67 ± 3.75) and reduced slightly over time (14.15 ± 0.13, 96 h p.i.). In addition, the ratios of tumor-to-heart (T/H: 14.28 ± 2.46), tumor-to-liver (T/L: 3.73 ± 1.21), tumor-to-kidney (T/K: 2.35 ± 0.61) increased more than two-fold from 24 h to 96 h p.i. The low uptakes of nontarget organs led to the high contrast of SPECT images. Compared with ^177^Lu-EB-FAPI-B1, the other radiotracers, especially ^177^Lu-EB-FAPI-B3 underperformed. It demonstrated higher uptakes in the kidneys (T/K: 0.64 ± 0.04) and liver (T/L: 0.75 ± 0.09), which resulted in low tumor uptake (T/M: 9.97 ± 1.23) at the stated time points **(Figure S18B)**.

On the basis of the observation in SPECT imaging, ^68^Ga-EB-FAPI-B1 was selected and compared with ^68^Ga-FAPI-02 with regard to U87MG tumor-targeting specificity and pharmacokinetic profile, by using PET imaging. As shown in **Figure S19A**, ^68^Ga-EB-FAPI-B1 was absorbed highly by heart and blood at 1 h p.i., while tumor uptake seemed relatively low due to high background intensity. Even so, the tumor uptake value of ^68^Ga-EB-FAPI-B1 (3.5 ± 0.22 %ID/g) was significantly higher than that of ^68^Ga-FAPI-02 (0.85 ± 0.21 %ID/g) (**Figure S19B**), respectively. Target specificity was assessed through simultaneous administration of unlabeled EB-FAPI-B1 as a competitor to compete with ^68^Ga-FAPI-02, the tumor uptake was suppressed significantly at 1 h p.i. The tumor uptake of ^68^Ga-FAPI-02 decreased more than half in the presence of the blocking agent EB-FAPI-B1. Furthermore, ^68^Ga-EB-FAPI-B1 also demonstrated a higher T/M ratio as compared with ^68^Ga-FAPI-02 (**Figure S19C**). These evidences suggested a strong affinity of ^68^Ga-EB-FAPI-B1 to tumor in PET imaging study.

### Biodistribution Study

**Figure 6:**
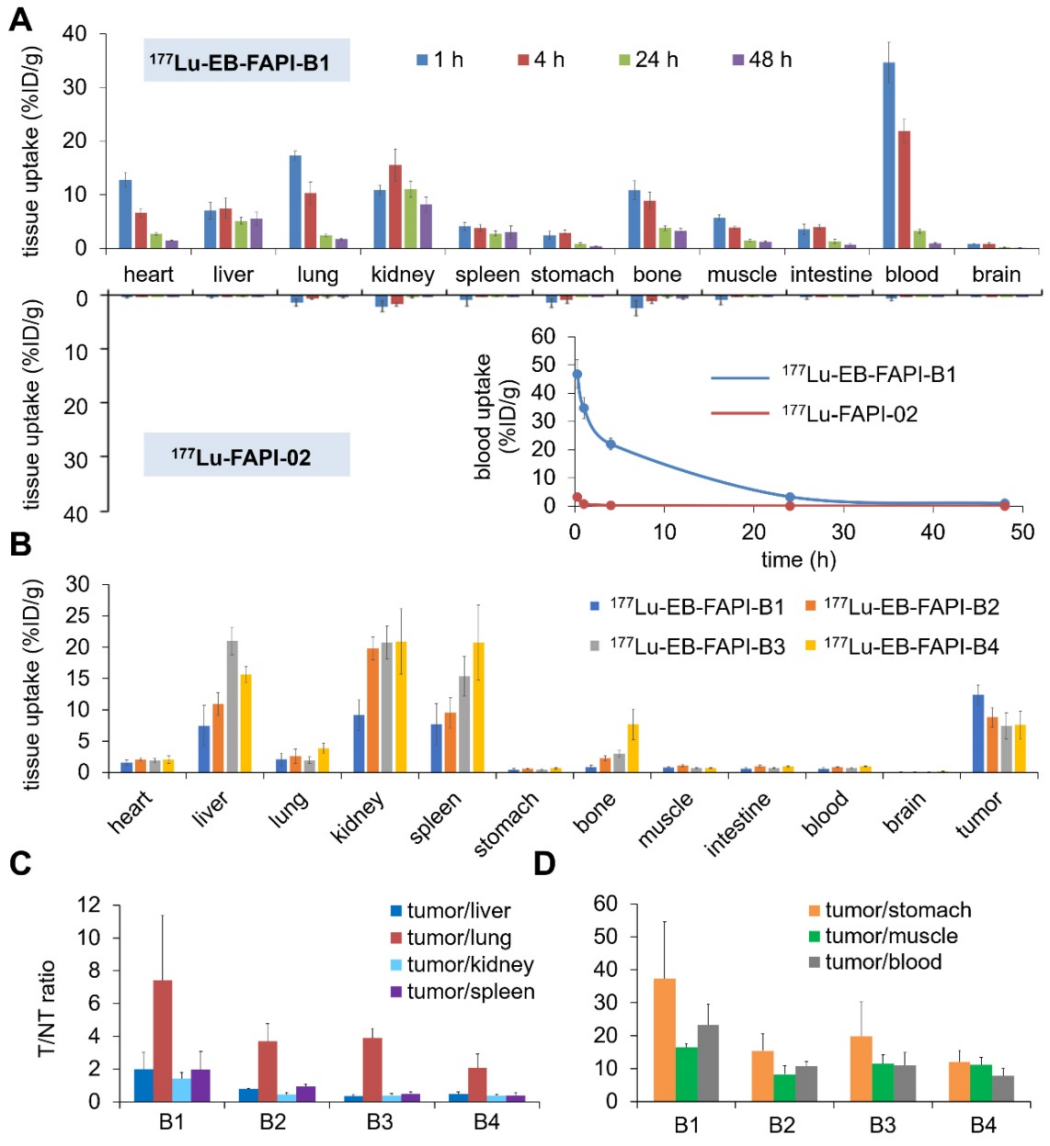
** (A)** Biodistribution studies and blood elimination curves of ^177^Lu-EB-FAPI-B1 and ^177^Lu-FAPI-02 in healthy ICR mice at different time points (n = 4/group); **(B)** Biodistribution study of ^177^Lu-EB-FAPI-B1, B2, B3 and B4 in U87MG tumor bearing mice at 96 h after injection (n = 3/group); **(C and D)** The ratios of tumor/nontarget tissues for ^177^Lu-EB-FAPI-B1, B2, B3 and B4 in U87MG tumor model at 96 h p.i.

The biodistribution results of ^177^Lu-FAPI-02 and ^177^Lu-EB-FAPI-B1, B2, B3, B4 in healthy ICR mice were shown in **Figure 6A** and **Figure S20**. At 0.25, 1 and 4 h p.i., for ^177^Lu-EB-FAPI-B1, B2, B3, B4, much higher blood radioactivity was observed than that of other tissues, indicating the primary distribution in the circulation system. For ^177^Lu-EB-FAPI-B1, blood uptake still sustained at 21.93 ± 2.13 %ID/g level at 4 h p.i. in spite of the decline of radioactivity in blood at all times. But the blood accumulation of ^177^Lu-FAPI-02 decreased to 0.23 ± 0.06 %ID/g at 4 h p.i., about 1% of ^177^Lu-EB-FAPI-B1 in the blood (**Figure 6A**), indicating the modification of FAPI-02 with EB could significantly prolong the blood circulation time in order to increase the probability of tumor uptake. Blood elimination curves of ^177^Lu-FAPI-02 and ^177^Lu-EB-FAPI-B1 shown in **Figure 6A** demonstrated the remarkably slow elimination rate for ^177^Lu-EB-FAPI-B1 during the first 24 h. The kidney uptake of ^177^Lu-EB-FAPI-B1 reached the highest values (16.38 ± 2.98 %ID/g) at 8 h p.i. and then declined to 8.13 ± 1.36 %ID/g at 48 h p.i. **(Figure S20A)**. Compared to ^177^Lu-EB-FAPI-B1, other radiotracers had a similar distribution pattern in ICR mice **(Figure S20B-D)**. The time-activity curves of ^177^Lu-EB-FAPI-B1, B2, B3, B4 in blood, kidneys and liver were obtained, respectively (**Figure S21**). These results demonstrated that ^177^Lu-EB-FAPI-B2, B3, B4 had higher uptakes in kidneys and liver compared with ^177^Lu-EB-FAPI-B1, which might eventually lead to higher toxicity *in vivo*.

Biodistribution results of ^177^Lu-EB-FAPI-B1, B2, B3, B4 in U87MG tumor-bearing mice were acquired through collecting tissues and *ex vivo* counting after mice were sacrificed at 96 h p.i. (**Figure 6B**). Tumor uptake of ^177^Lu-EB-FAPI-B1 still retained high value at 96 h p.i. (12.42 ± 1.54 %ID/g) that exceeded the other organs. On the contrary, higher uptakes of the other radiotracers were observed in the liver, kidneys and spleen, than that in the tumor at 96 h p.i. For example, uptakes of ^177^Lu-EB-FAPI-B3 in the liver (20.99 ± 2.18 %ID/g), kidneys (20.73 ± 2.63 %ID/g) and spleen (15.36 ± 3.14 %ID/g) far exceeded the tumor uptake of ^177^Lu-EB-FAPI-B3 (7.42 ± 2.09 %ID/g). As shown in **Figure 6C-D**, ^177^Lu-EB-FAPI-B1 had higher tumor/normal tissue ratios, especially tumor/stomach, tumor/muscle and tumor/blood, than the other compounds. The results obtained from the biodistribution were generally in agreement with the foregoing observations from SPECT imaging. These results confirmed that ^177^Lu-EB-FAPI-B1 could be a great lead compound for radioligand therapy.

### Radioligand Therapy Study and Histopathological Analysis

**Figure 7:**
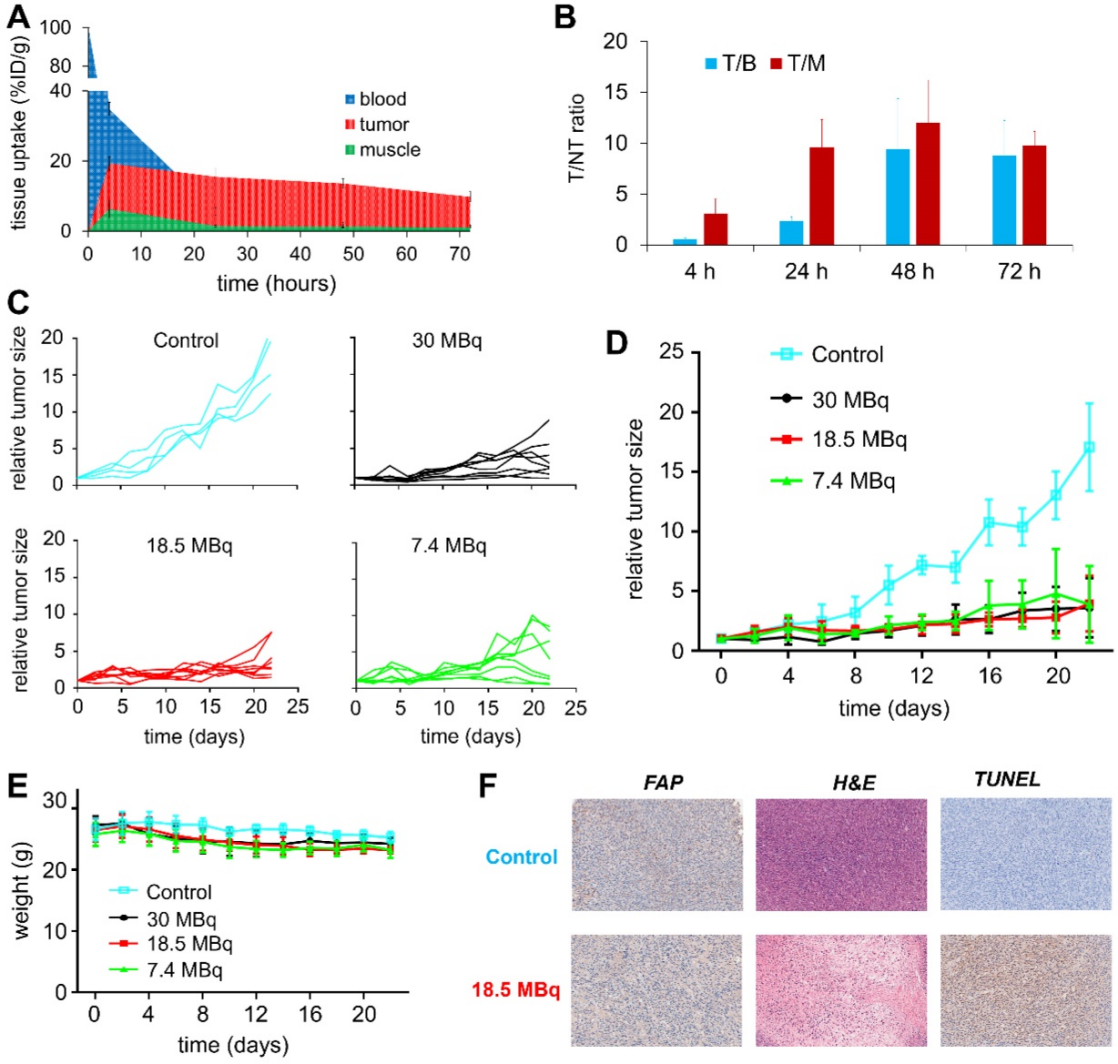
** (A)** Tissue uptake and variation trend of ^177^Lu-EB-FAPI-B1 in the blood, tumor and muscle at different times after injection; **(B)** T/NT ratios of ^177^Lu-EB-FAPI-B1 *in vivo* at 4, 24, 48 and 72 h p.i.; **(C)** Changes in the relative tumor size of individual mice after treated in different dosages of ^177^Lu-EB-FAPI-B1 (n = 4 mice for control, n = 8 mice for the other groups); **(D)** The tumor growth curves after treatment with different dosages of ^177^Lu-EB-FAPI-B1; **(E)** Mice body weight change after treatment; **(F)** IHC staining of FAP expression, H&E staining and TUNEL staining were performed in excised U87MG tumor.

To evaluate the therapeutic efficacy of ^177^Lu-EB-FAPI-B1, U87MG tumor-bearing mice were used for the radioligand therapy study. The time-activity curves of ^177^Lu-EB-FAPI-B1 in blood, tumor and muscle were shown in **Figure 7A**, and T/NT ratios of ^177^Lu-EB-FAPI-B1 in U87MG tumor mice at 4, 24, 48 and 72 h p.i. were calculated (**Figure 7B**). Compared to the saline group, the groups treated by single dose of 30 MBq, 18.5 MBq or 7.4 MBq of ^177^Lu-EB-FAPI-B1 showed noteworthy tumor growth delay **(Figure 7C-D)**. Tumor growth was inhibited by all three doses of ^177^Lu-EB-FAPI-B1, demonstrated by the relative tumor size reached no more than 5 throughout the measurement from day 0 to day 22, whereas the relative tumor size in the saline group reached more than 15. In addition, slight weight loss was observed for all the treatment groups, but it fluctuated within the normal range (**Figure 7E**).

To support the findings, FAP staining to evaluate fibroblast activation protein expression level, TUNEL staining to determine DNA damage, H&E staining to assess the degree of tissue destruction were conducted on histological slides of excised tumors. As shown in **Figure 7F**, the high expression of FAP was observed both in the saline group and the 18.5 MBq ^177^Lu-EB-FAPI-B1 group. H&E staining showed large necrotic areas in the treatment groups compared to saline group, which demonstrated the distinct therapeutic efficacy. In comparison with the saline group, 18.5 MBq ^177^Lu-EB-FAPI-B1 group showed remarkably more cell apoptosis, as displayed by TUNEL staining. Safety evaluation was performed by H&E staining of major organs, the results showed negligible side effect after treatment with ^177^Lu-EB-FAPI-B1 (**Figure S22**).

## Discussion

One of the major considerations for a rational drug design is its PK and PD in the blood. A drug with shorter blood circulation half-life leads to less accumulation at a target organ, indicating the need for higher or more frequent dosages, both of which may increase the possibility of undesirable side effects. The reversible binding of EB to albumin has been documented in previous studies 30, 31, and EB-RGD, EB-TATE and EB-PSMA conjugates have been developed to improve PK and PD of RGD, TATE and PSMA, which were successfully used for radioligand therapy 21-23. In this study, we modified FAPI-02-based structure with EB moiety for the first time to synthesize a series of radiopharmaceuticals that are more efficacious than FAPI-02 for FAP targeted radiotherapy. The series of compounds of EB-FAPI-B1, B2, B3, B4 were synthesized and radiolabeled with ^177^Lu. These radiotracers showed good stability and high FAP binding affinity *in vitro*. SPECT imaging and biodistribution study of the probes demonstrated that significant tumor accumulation was still prominent at 96 h p.i.

PEGylation is also a widely used strategy for improving the PK of radiotracers 32. We hypothesized that further modifying the FAPI-based structure with PEG may enhance the hydrophilicity and improve the PK and PD properties *in vivo*, thus decrease the background and increase the tumor accumulation. However, the results did not find better tumor uptake and distribution *in vivo* with PEG modification. For ^177^Lu-EB-FAPI-B1, the radioligand without PEG modification, higher tumor uptake and lower normal tissue signal were observed than that of B2, B3 and B4. It showed that PEGylation slightly enhances hydrophilicity, obviously increases normal tissues uptake, especially kidneys and liver, but decreases desirable tumor uptake. This can be possibly explained by “PEG dilemma”, an issue that is often described as hindered cellular uptake of macromolecules and thus compromised effective drug delivery to target cancer cells caused by PEGylation 33. Therefore, EB-FAPI-Bn compounds modified with PEG might be against the performance improvement of FAP targeting and delivery. Another explanation is that the primary regions of intermolecular binding between EB-FAPI-Bn and FAP situated in the hydrophobic cavities from molecular docking modeling, it could be that hydrophobicity is an important factor for FAP protein binding. Therefore, longer PEG might cause worse PD and PK properties for FAPI radiopharmaceuticals.

Although PEGylation did not have the expected effect in improving PK of drugs, ^177^Lu-EB-FAPI-B1 demonstrated great potential to be applied clinically for a wide spectrum of tumor theranostics. FAPI-02 was modified by functional chemical group (EB) rather than by subtle chemical derivatizations, allowing a massive increase in FAPI-02 performance in regards to PK and PD properties. During circulation, EB-FAPI-02 will bind to albumin in a dynamic way, and will be released from albumin when in proximity with FAP in the tumor stroma. The complexes will then function as drug reservoir that can enhance FAPI-02 distribution and availability. This is confirmed by significant uptake in the heart and blood during the initial 4 h p.i., greatly improved tumor uptake compared with FAPI-02. Because of this enhancement, the tumor targeting specificity for FAPI-02 also dramatically improved, as demonstrated by the sustained tumor retention even at 96 h p.i., and low normal tissue uptake, resulting in high tumor-to-background ratio, which is conducive for cancer therapy to a great extent.

In previous studies, several FAPI related radiopharmaceuticals have been clinically used for cancer therapy. For example, ^177^Lu-FAPI-46 and ^225^Ac-FAPI-46 for the treatment of pancreatic carcinoma 34, ^177^Lu-FAPI-04 for the therapy of triple-negative breast cancer and metastatic ovarian carcinosarcoma 35, ^90^Y-FAPI-04 for treating a final stage breast cancer patient with bone metastases 10. Compared with clinically established peptide-based radionuclide therapies, ^177^Lu-FAPI-04 has a significantly lower absorbed radiation dose to major organs. Bone marrow is the most radiosensitive tissue, and as the dose-limiting organ could tolerate the accumulative radioactivity up to 50 GBq. The mean administered activity of ^177^Lu-FAPI-04 in patients was 267.5 ± 8.6 MBq, that is far below the limiting value, but the absorbed dose in the tumor region is also unsatisfactory 35. To optimize the therapeutic efficacy, Xu *et al.* investigated the albumin binder conjugated FAPI-04 radiopharmaceuticals for cancer therapy and achieved reasonable outcome 36. Instead of using FAPI-04, we developed a more outstanding radiopharmaceutical based on FAPI-02, ^177^Lu-EB-FAPI-B1, which has some obvious advantages: firstly, compared to SPECT imaging of ^177^Lu-TEFAPI-07 (FAPI-04 modified with EB moiety), the lead compound ^177^Lu-EB-FAPI-B1 had lower kidney uptake and significantly higher tumor uptake from 24 h to 96 h p.i. SPECT imaging showed the superior T/NT contrast for ^177^Lu-EB-FAPI-B1 (**Figure 5A**). The high tumor uptake and low normal tissue uptake would improve the therapeutic efficacy and reduce the side effects in cancer therapy. Secondly, compared with FAPI-02, FAPI-04 showed higher affinity to FAP, slower excretion *in vitro* and a higher standardized uptake value in tumor-bearing mice 10. It is also of note that although the treatment protocol of ^177^Lu-TEFAPI-07 in pancreatic cancer models achieved certain therapeutic effect, therapy protocols of three cycles increased the side effects and treatment expense. ^177^Lu-EB-FAPI-B1 with single dose significantly inhibited tumor growth in our study, thus the balance of treatment cycles and dosages need to be considered.

During radiation treatment planning, increasing the dosage is considered a passive way to improve the outcome of cancer treatment, with the therapeutic efficacy indisputably being conditioned by the T/NT ratio 37. In this study three doses (7.4, 18.5, and 30 MBq) of radiotracers generated comparably similar therapeutic efficacy in terms of tumor growth inhibition. It is also worthwhile to note that, as the dose was increased from 7.4 MBq to 30 MBq, the therapeutic effect of ^177^Lu-EB-FAPI-B1 was not improved. It may suggest that the superfluous radiotracers is not able to be effectively taken up by the tumor but rather end up to be accumulated in the normal tissue or excreted from the circulation. Moreover, we observed slight body weight loss, thus the side effect may not be completely ignored. Therefore, H&E staining studies of major organs including heart, liver, spleen, lung, kidneys were performed and no distinct tissue damage was found (**Figure. S22**). Follow-up studies are suggested to consider using multiple cycles of administration even lower dosage for tumor inhibition studies.

## Conclusion

In this research, we developed a series of EB conjugated FAPI-02-based radiotracers for tumor theranostics. ^177^Lu-EB-FAPI-B1 demonstrated greatly improved binding affinity and FAP targeting specificity *in vitro*. Massively enhanced tumor retention and decreased normal tissue uptake were also observed. Most importantly and excitingly, ^177^Lu-EB-FAPI-B1 showed remarkable tumor growth suppression effect on U87MG tumor-bearing mice and negligible side effect, suggesting that the EB modified FAPI structures can be applied as promising drugs for radioligand therapy of cancer.

## Supplementary Material

Supplementary materials and methods, figures and tables.
