# Supplementary material for: Evans blue-modified radiolabeled fibroblast activation protein inhibitor as long-acting cancer therapeutics
Source: Theranostics. 2022 Jan 1;12(1):422–33. doi: 10.7150/thno.68182 (PMC8690933; doi:10.7150/thno.68182)
Supplement: Supplementary file 1 — Supplementary materials and methods, figures and tables. [file thnov12p0422s1.pdf]

Supplementary materials

Evans blue-modified radiolabeled fibroblast activation protein inhibitor as long-acting cancer therapeutics

Xuejun Wen[1#], Pengfei Xu[2#], Mengqi Shi[3,4#], Jia Liu[1], Xinying Zeng[1], Yiren Zhang[1], Changrong Shi[1], Jingchao Li[1], Zhide Guo[1]*, Xianzhong Zhang[1]*, Pek-Lan Khong[3,5*], Xiaoyuan Chen[3,4,5,6]*

[3] Department of Diagnostic Radiology, Yong Loo Lin School of Medicine, National University of Singapore, 119074, Singapore

[4] Department of Chemical and Biomolecular Engineering, and Department of Biomedical Engineering, Faculty of Engineering, National University of Singapore, Singapore, 117575, Singapore

[4] Nanomedicine Translational Research Program, NUS Center for Nanomedicine, Yong Loo Lin School of Medicine, National University of Singapore, Singapore 117597, Singapore

[5] Clinical Imaging Research Centre, Centre for Translational Medicine, Yong Loo Lin School of Medicine, National University of Singapore, Singapore 117599, Singapore

[#]These authors contributed equally to this work.

***Correspondence to:**

Zhide Guo, E-mail: gzd666888@xmu.edu.cn;

Xianzhong Zhang, E-mail: zhangxzh@xmu.edu.cn;

Pek-Lan Khong, E-mail: dnrkpl@nus.edu.sg;

Xiaoyuan Chen, E-mail: chen.shawn@nus.edu.sg

Materials and Characterization

General

All chemical reagents were obtained from the commercial suppliers and used without further purification. The ^{1}H and ^{13}C NMR spectra were acquired on Bruker magnetic resonance spectrometers. MALDITOF-MS spectra were obtained by an AB SCIEX 4700 TOF/TOF System.

Analytical high-performance liquid chromatography (HPLC) was done on symmetry C-18 columns from YMC (3 µm, 150 × 4.6 mm i.d.) using 0.1% trifluoroacetic acid (TFA)/H_2O (solvent A) and 0.1% trifluoroacetic acid (TFA) CH_3CN (solvent B) at flow rate of 1 mL/min. Gradient elution was performed as follows: 10% of B, 0-3 min;10-90% of B, 3-15 min; 90% of B, 15-16 min; 90-10% of B, 16-18 min; 10% of B, 18-25 min. The ultraviolet (UV) absorbance was monitored at 254 nm.

Compounds were purified on Interchim puriFlash 4250 system equipped with C-18 columns from Waters Xbrige (5 µm, 250 × 20 mm i.d.) at flow rate of 12 mL/min. Gradient elution was performed as follows: 5% of B, 0-10 min; 5-70% of B, 10-40 min; 70% of B, 40-60 min. The UV absorbance was monitored at 254 nm.

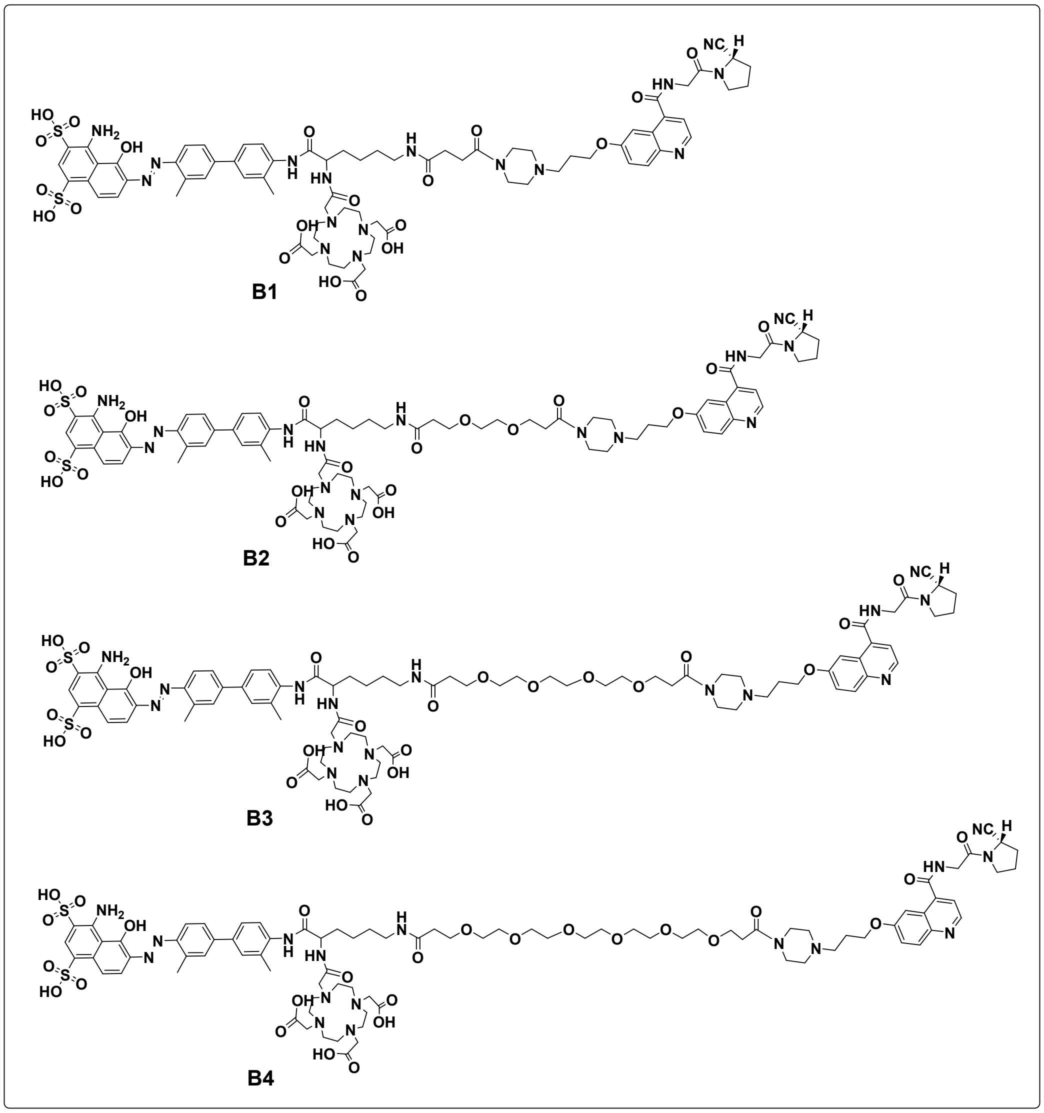

Chemical structures of EB-FAPI-B1, EB-FAPI-B2, EB-FAPI-B3 and EB-FAPI-B4.

Chemistry

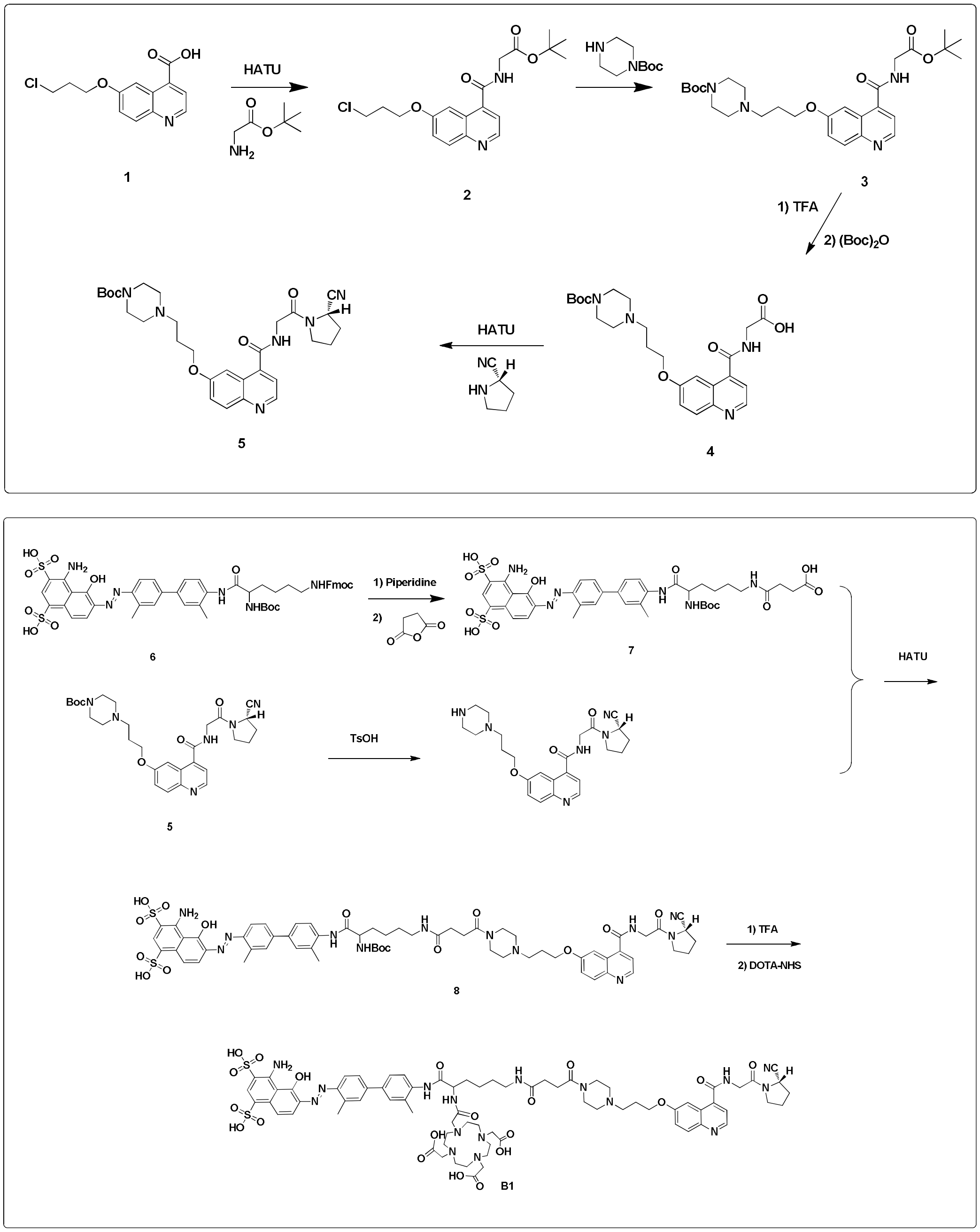

Synthetic route for compound EB-FAPI-B1

Synthesis of Compound 2. Compound 1 was prepared according to previous work. (DOI: 10.2967/jnumed.118.210435) To a solution of **Compound 1** (2.65 g, 10 mmol), N,N-diisopropylethylamine (1.52 g, 12 mmol) and O-(7-azabenzotriazol-1-yl)-N,N,N',N'-tetramethyluronium hexafluorophosphate (3.8 g, 10 mmol) in DMF (20 mL)

was added compound **1** (1.43 g, 11 mmol). The reaction mixture was stirred at room temperature. The reaction was monitored by TLC and it was completed in 12 hours. The reaction mixture was concentrated in vacuo and the residue was purified by silica gel chromatography (DCM:MeOH = 50:1 v/v) to give **Compound 2** (3.17g, 84% yield). ESI: m/z 379.11 $(M+H)^+$. ^1H NMR (400 MHz, $CDCl_3$) δ 8.70 (d, *J* = 4.4 Hz, 1H), 7.97 (d, *J* = 9.2 Hz, 1H), 7.67 (d, *J* = 2.7 Hz, 1H), 7.41 (d, *J* = 4.4 Hz, 1H), 7.36 (dd, *J* = 9.2, 2.7 Hz, 1H), 4.27 (t, *J* = 5.8 Hz, 2H), 4.22 (d, *J* = 5.4 Hz, 2H), 3.78 (t, *J* = 6.3 Hz, 1H), 3.64 (t, *J* = 6.5 Hz, 0H), 2.43 – 2.24 (m, 2H), 1.52 (s, 9H).

Synthesis of Compound 3. Compound 2 (1.89 g, 5 mmol), N-tert-butoxycarbonylpiperazine (2.79 g, 15 mmol) and potassium iodide (0.42 g, 2.5 μmol) were dissolved in 20 mL DMF. The reaction was shaken at 60 ºC for 12 h. The reaction mixture was concentrated in vacuo and the residue was purified by silica gel chromatography (DCM:MeOH = 50:1 v/v) to give **Compound 3** (1.68g, 64% yield). ESI: m/z 529.10 $(M+H)^+$. ^1H NMR (400 MHz, $CDCl_3$) δ 8.78 – 8.67 (m, 1H), 7.97 (t, *J* = 8.6 Hz, 1H), 7.65 (t, *J* = 6.7 Hz, 1H), 7.43 (d, *J* = 4.4 Hz, 1H), 7.37 (dd, *J* = 9.2, 2.7 Hz, 1H), 6.87 (t, *J* = 5.3 Hz, 1H), 4.21 (d, *J* = 5.4 Hz, 2H), 4.17 (t, *J* = 6.2 Hz, 2H), 3.48 – 3.40 (m, 4H), 2.61 – 2.53 (m, 2H), 2.47 – 2.39 (m, 4H), 2.09 – 1.97 (m, 2H), 1.52 (s, 9H), 1.46 (s, 9H). ^{13}C NMR (101 MHz, $CDCl_3$) δ 168.77, 167.75, 158.01, 154.73, 146.95, 144.90, 139.61, 131.07, 125.66, 123.28, 118.97, 103.54, 82.70, 79.65, 66.43, 55.16, 53.03, 42.34, 31.22, 28.42, 28.09.

Synthesis of Compound 4. To a solution of **Compound 3** (530 mg, 1 mmol) in 9 mL was added TFA (1 mL). The mixture was stirred at room temperature. The reaction was monitored by HPLC and it was completed in 4 h. The reaction mixture was concentrated in vacuo and the residue was resolved in 5 mL DMF. Then, di-tert butyl dicarbonate (654 mg, 3 mmol) and N,N-diisopropylethylamineand (760 mg, 6 mmol) were added. The reaction was monitored by HPLC analysis and it was completed in 5 hours. The reaction mixture was concentrated in vacuo and the residue was purified by silica gel chromatography (DCM:MeOH = 10:1 v/v) to give **Compound 4** (340 mg, 72% yield) ESI: m/z 473.10 $(M+H)^+$.

Synthesis of Compound 5. To a solution of **Compound 4** (230 mg, 0.5 mmol), N,N-diisopropylethylamineand (129 mg, 1 mmol) and O-(7-Azabenzotriazol-1-yl)-N,N,N',N'-tetramethyluronium hexafluorophosphate (190 mg, 0.5 mmol) in DMF (5 mL) was added (S)-pyrrolidine-2-carbonitrile (50 mg, 0.5 mmol). The reaction mixture was stirred at room temperature for 6 h. DMF was removed under high vacuum and the residue was purified by silica gel chromatography (DCM:MeOH = 10:1 v/v) to give **Compound 5** (222 mg, 81% yield) ESI: m/z 551.12 $(M+H)^+$. ^1H NMR (400 MHz, $CDCl_3$) δ 8.80 (d, *J* = 4.4 Hz, 1H), 8.03 (d, *J* = 9.2 Hz, 1H), 7.70 (d, *J* = 2.6 Hz, 1H), 7.50 (d, *J* = 4.4 Hz, 1H), 7.39 (dd, *J* = 9.2, 2.7 Hz, 1H), 4.82 – 4.73 (m, 1H), 4.35 (qd, *J* = 17.7, 4.6 Hz, 2H), 4.22 – 4.16 (m, 2H), 3.79 – 3.66 (m, 1H), 3.59 – 3.51 (m, 1H), 3.47 (s, 3H), 2.64 (t, *J* = 7.1 Hz, 2H), 2.50 (s, 3H), 2.41 – 2.19 (m, 4H), 2.07 (dt, *J* = 13.5, 6.6 Hz, 2H), 1.46 (s, 8H). ^{13}C NMR (101 MHz, $CDCl_3$) δ 167.81, 167.07, 158.05, 154.70, 147.06, 145.03, 139.07, 131.23, 125.64, 123.29, 119.24, 117.91, 103.51, 79.73, 66.41, 55.17, 53.02, 46.71, 45.62, 42.37, 31.24, 29.88, 28.42, 26.34, 25.12.

Synthesis of Compound 7. Compound 6 was prepared according to previous report. (DOI: 10.2967/jnumed.116.182097) Deprotection of Fmoc was done at RT using piperidine (20% [v/v]). The reaction mixture was stirred for 1 h, then DMF was removed under high vacuum. The residue was redissolved in methanol-H_2O (2:1) and purified on a Interchim puriFlash 4250 system. MaldiTOF-MS analysis confirmed mass of 769.661 $[M-H]^-$ with an isolated yield of 67%. Succinic anhydride (500 mg, 5 mmol) in 5 mL DMF was added to the Fmoc de-protected compound 6 intermediate (0.5 mmol, 375 mg), followed by addition of 10 mmol DIPEA. The mixture was stirred at RT for 12 h until analytical HPLC showed complete conversion to **compound 7**. Then, DMF was removed under high vacuum. The residue was re-dissolved in methanol-H_2O (2:1) and purified on a Interchim puriFlash 4250 system (278 mg, 64% yield).

Synthesis of Compound 8. Compound 5 (110 mg, 0.2 mmol) and 4-methylbenzenesulfonic acid monohydrate (380 mg, 2 mmol) were dissolved in 10 mL acetonitrile. The reaction was shaken at 45 °C overnight. Completion of deprotection was monitored by HPLC. Acetonitrile was removed and the residue was resolved in DMF. Then, **compound 7** (174 mg, 0.2 mmol), N,N-diisopropylethylamineand (129

mg, 1 mmol) and O-(7-azabenzotriazol-1-yl)-N,N,N',N'-tetramethyluronium hexafluorophosphate (76 mg, 0.2 mmol) were added. The reaction mixture was stirred at room temperature for 6 h. DMF was removed under high vacuum and the residue was re-dissolved in methanol-H_2O (2:1) and purified on a Interchim puriFlash 4250 system (210 mg, 81% yield).

Synthesis of Compound B1. De-protection of BOC groups of **compound 8** (26 mg, 0.02 mmol) were removed by 10% TFA in DCM (v/v) for 1 h at RT. Then, after the TFA and DCM were removed by a flow of argon, the residue was reacted with 1.2 eq of DOTA-NHS ester and 8 eq of DIPEA in DMF. After stirring at RT for 2-3 h, H_2O was added, and the reaction mixture was purified on a Interchim puriFlash 4250 system to give the desired product (23 mg, 73% yield). MALDI-TOF [Calcd. for $C_{74}H_{93}N_{16}O_{20}S_2^+$: 1589.21 found: m/z 1589.34].

Synthesis of Compounds B2, B3 and B4. Compounds B2, B3 and B4 were synthesized according to the general procedure described above.

Supplementary Figures

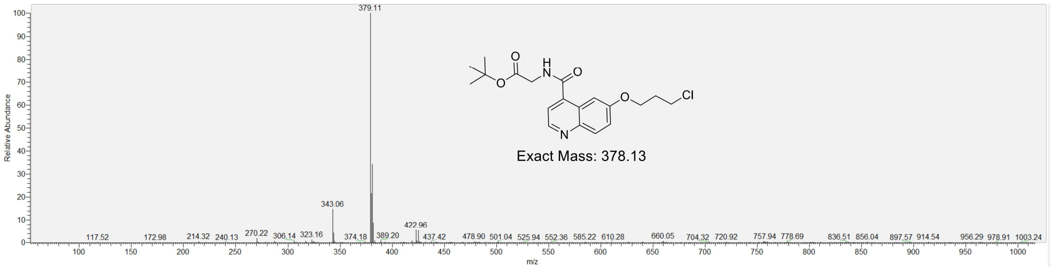

Figure S1. MS spectroscopy of compound 2.

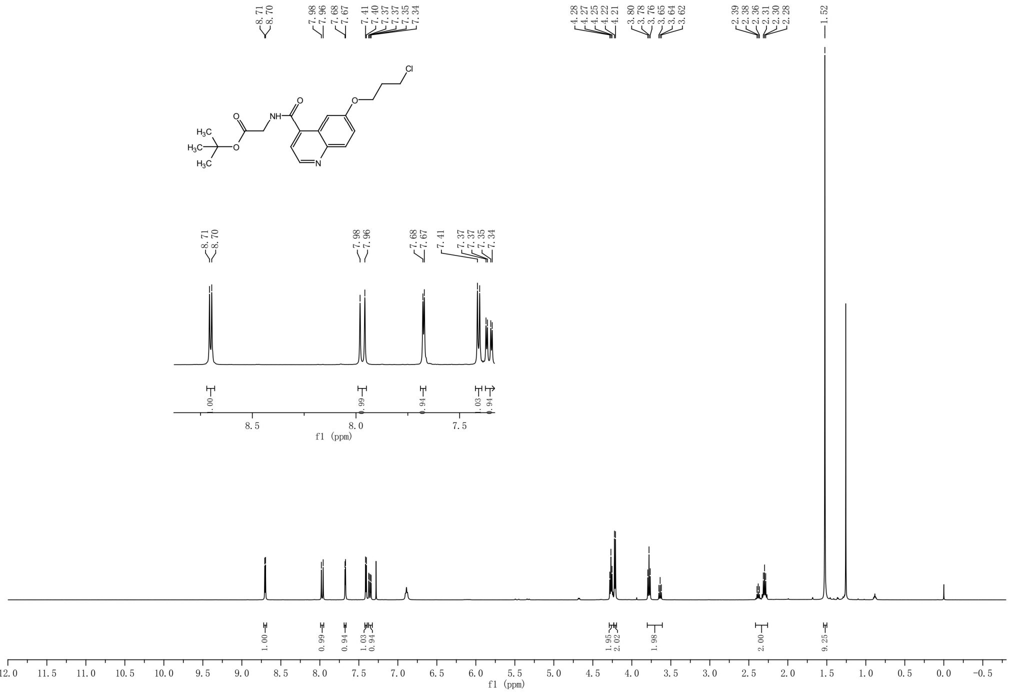

Figure S2. ^{1}H NMR spectroscopy of compound 2.

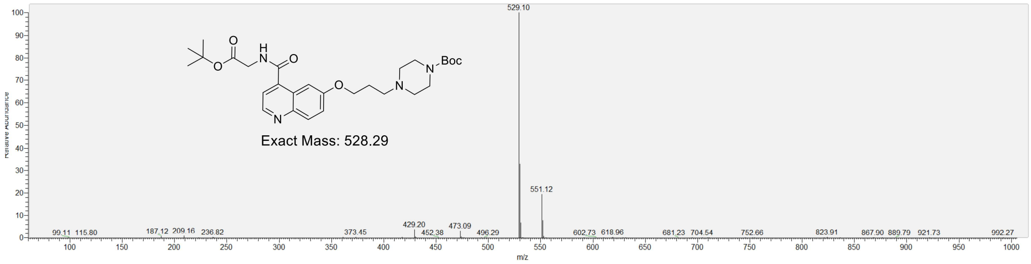

Figure S3. MS spectroscopy of compound 3.

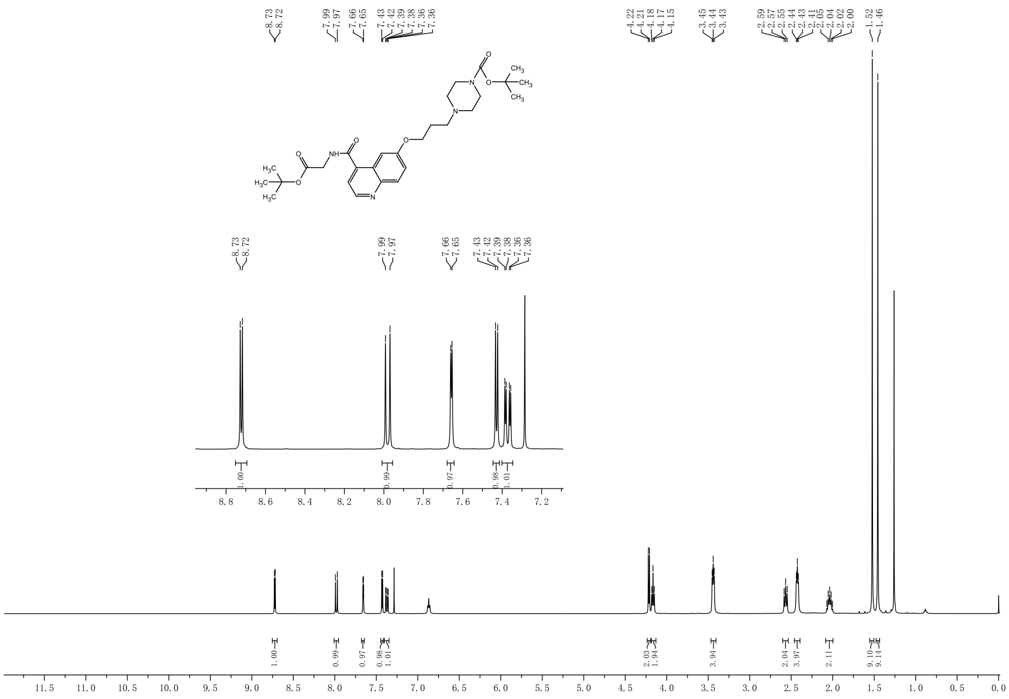

Figure S4. ^{1}H NMR spectroscopy of compound 3.

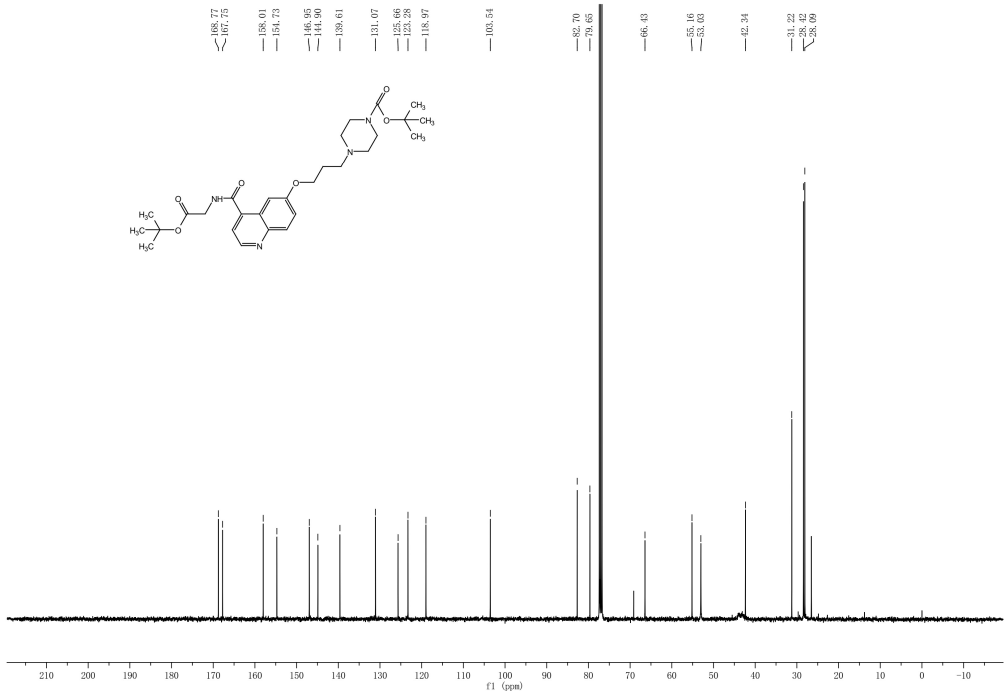

Figure S5. ^{13}C NMR spectroscopy of compound 3.

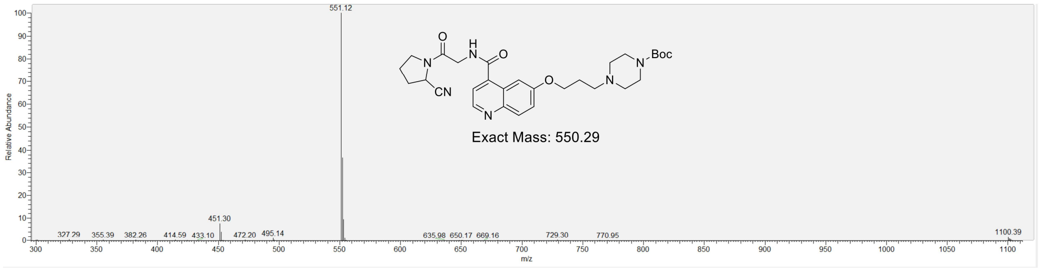

Figure S6. MS spectroscopy of compound 5.

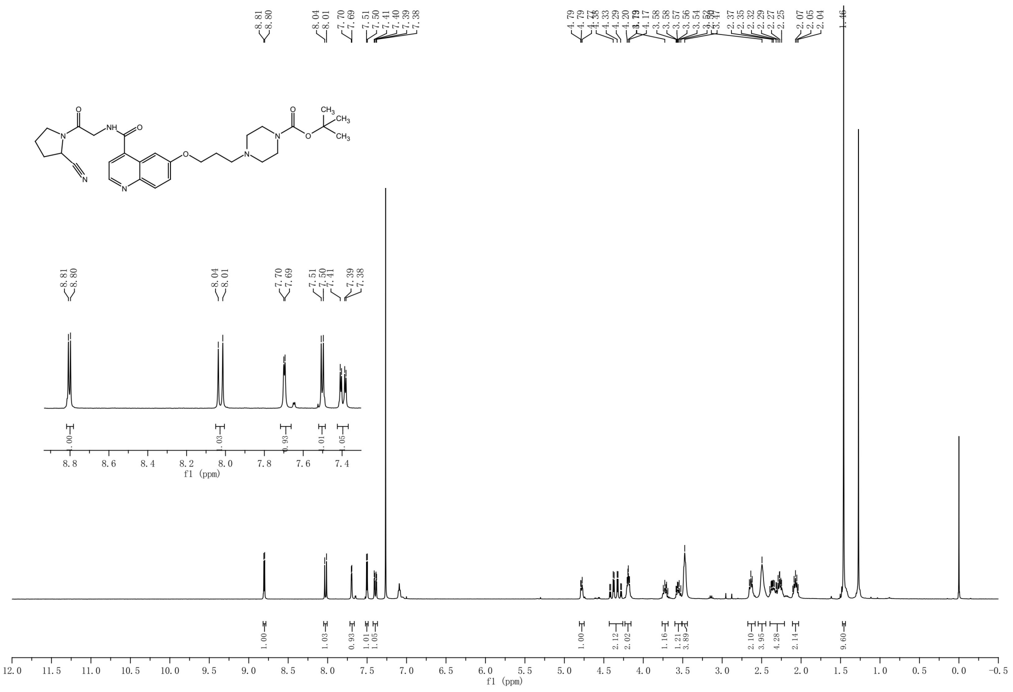

Figure S7. ^{1}H NMR spectroscopy of compound 5.

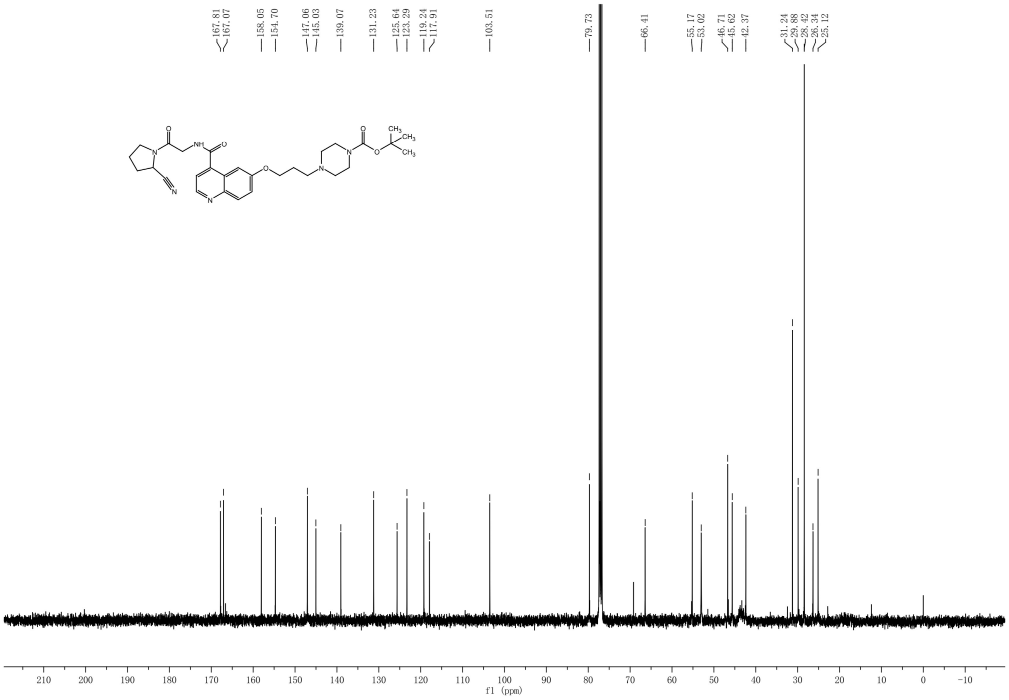

Figure S8. ^{13}C NMR spectroscopy of compound 5.

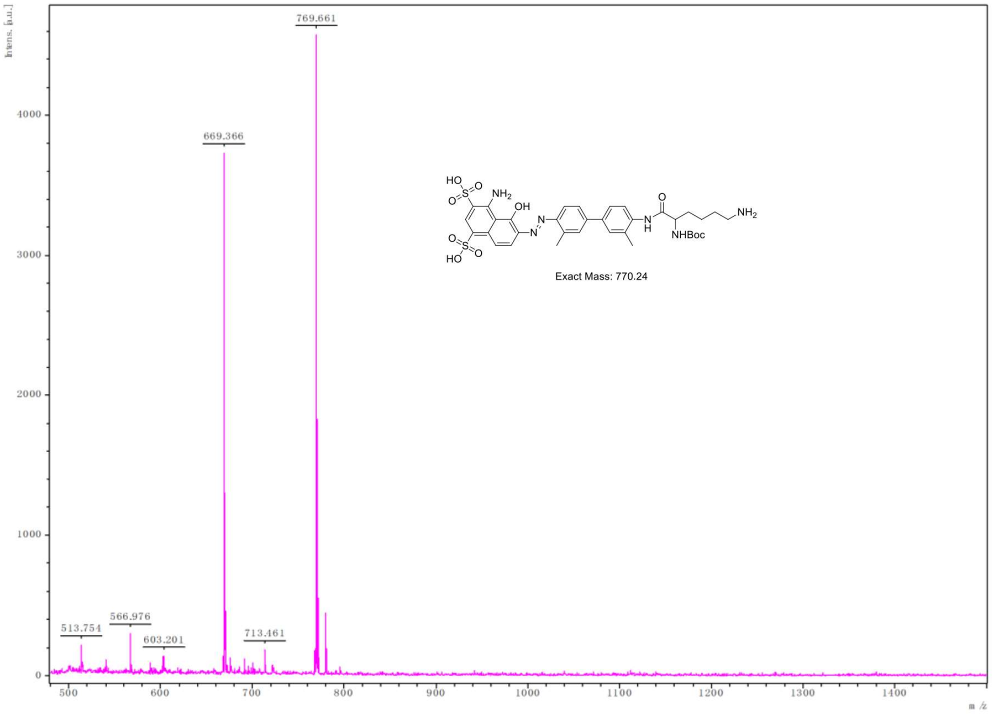

Figure S9. MALDI-TOF-MS measurement of FMOC de-protected compound 6.

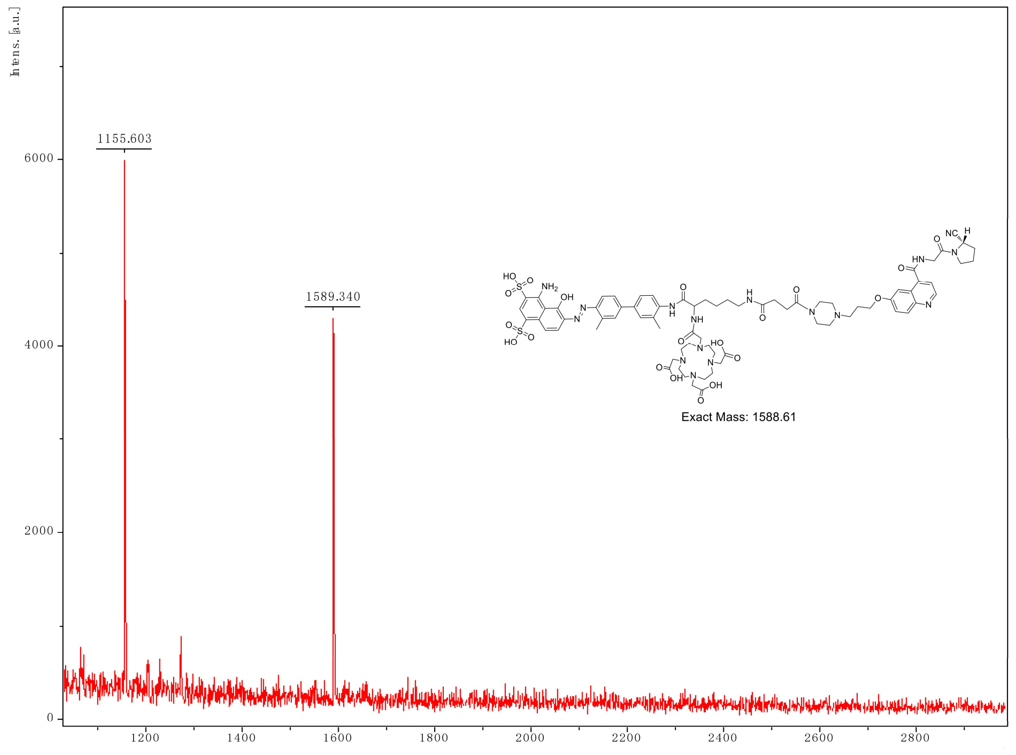

Figure S10. MALDI-TOF-MS measurement of compound B1.

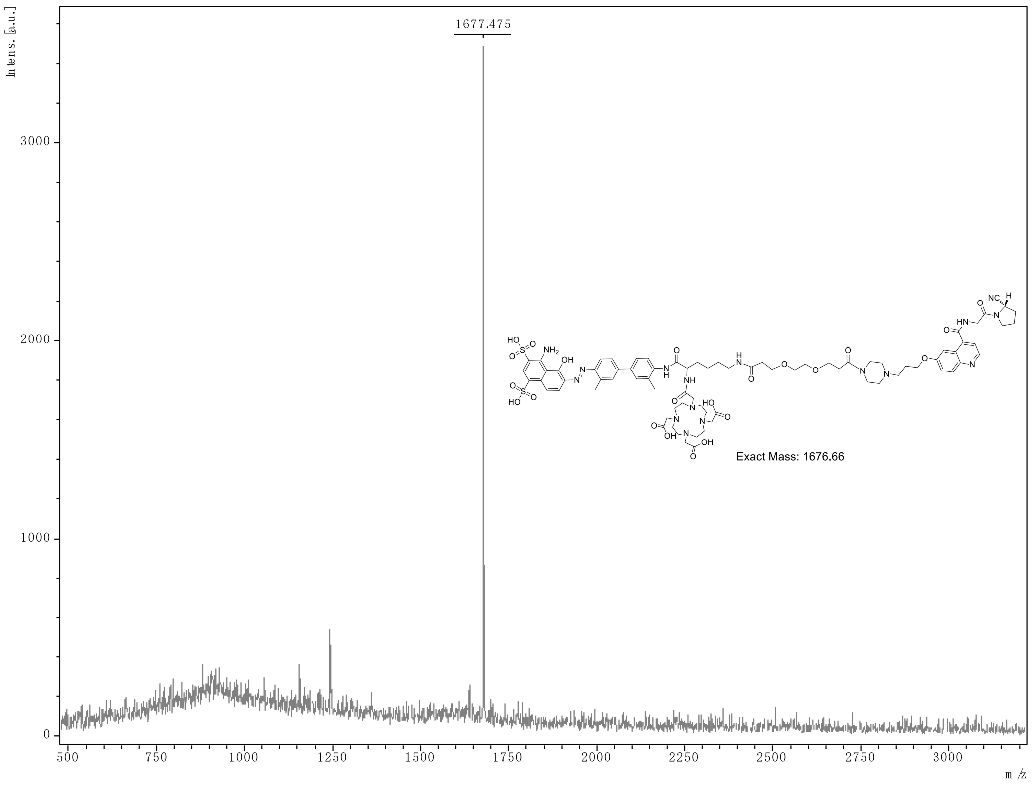

Figure S11. MALDI-TOF-MS measurement of compound B2.

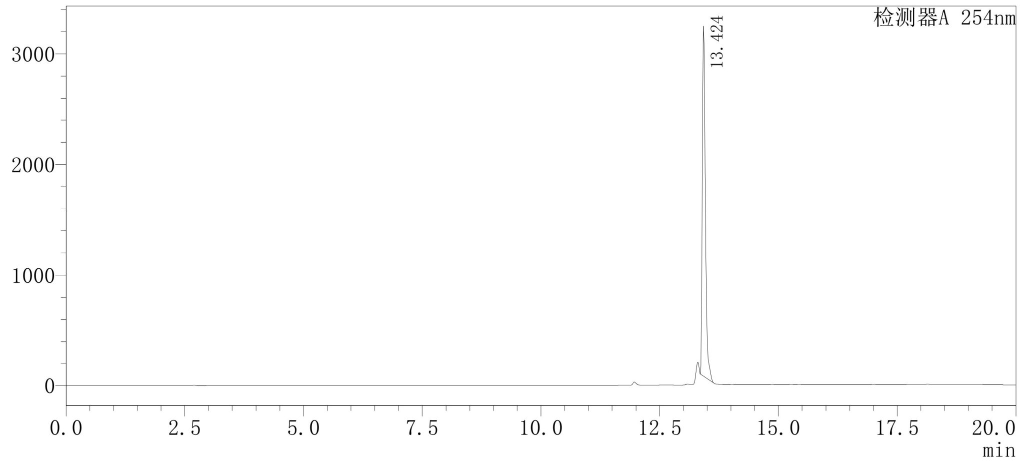

Figure S12. HPLC analysis of compound 7.

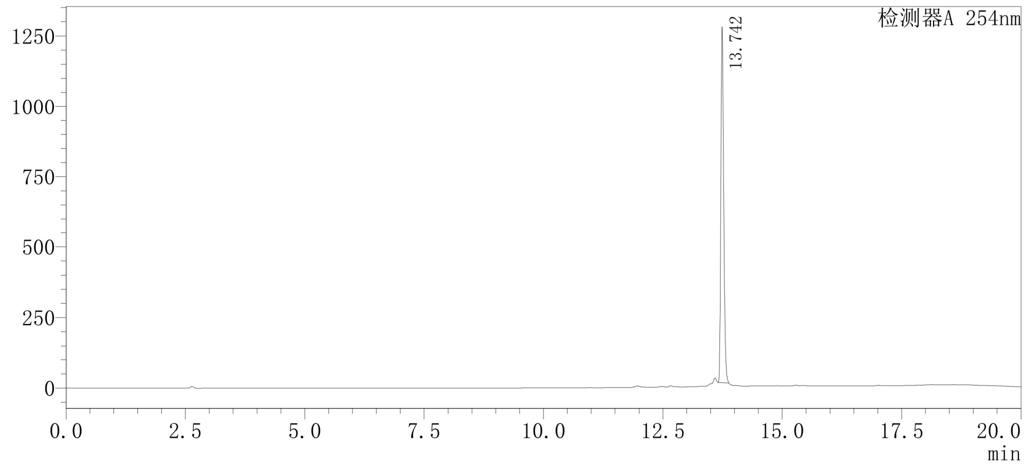

Figure S13. HPLC analysis of compound 8.

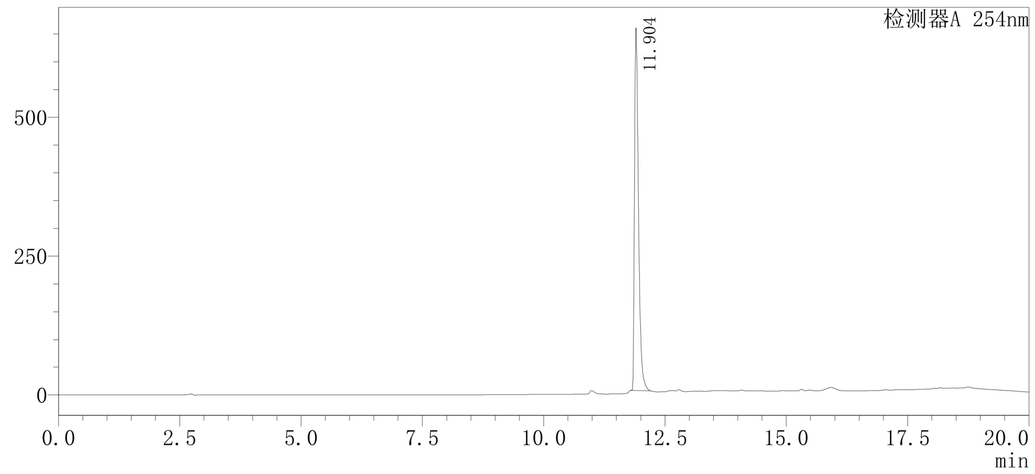

Figure S14. HPLC analysis of compound B1.

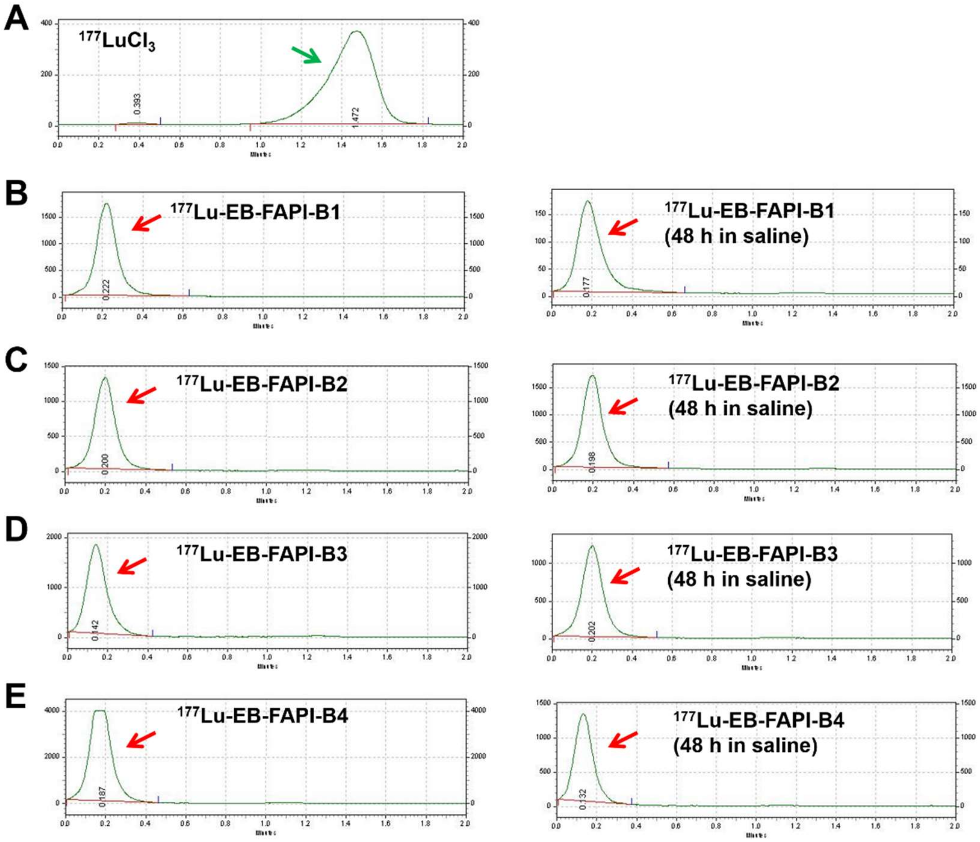

Figure S15. Radiochemical purity and stability of ^{177}Lu-EB-FAPI-B1 after incubation with saline for 48 h were measured by TLC. ^{177}Lu-EB-FAPI-B1 stayed at the point of origin (retention time: 0-0.2 min). $^{177}LuCl_3$ had retention time of 1.5-1.6 min.

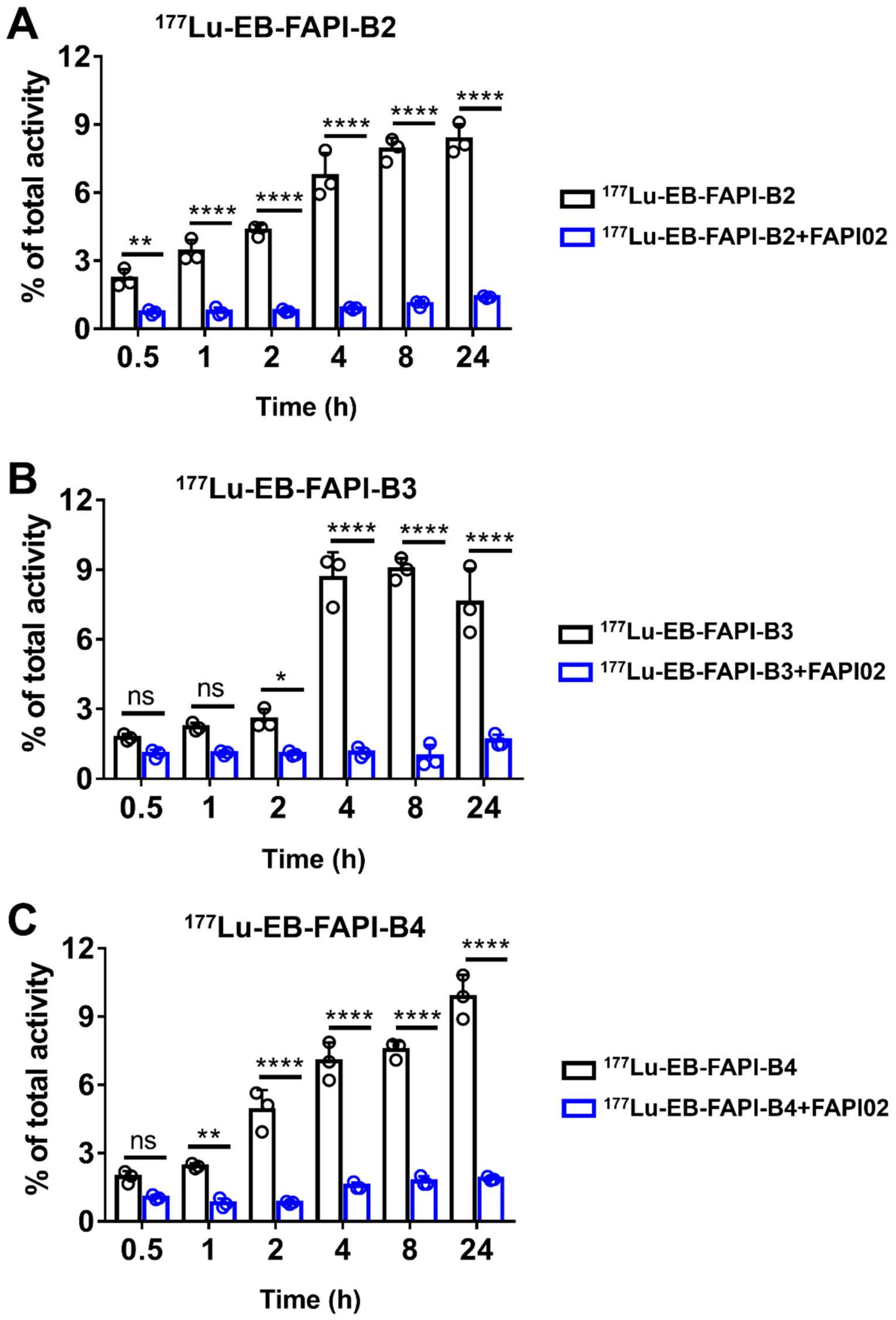

Figure S16. Cellular uptake assays of ^{177}Lu-EB-FAPI-B2 **(A)**, B3 **(B)**, B4 **(C)** and their blocking experiments by FAPI-02 in U87MG tumor cells.

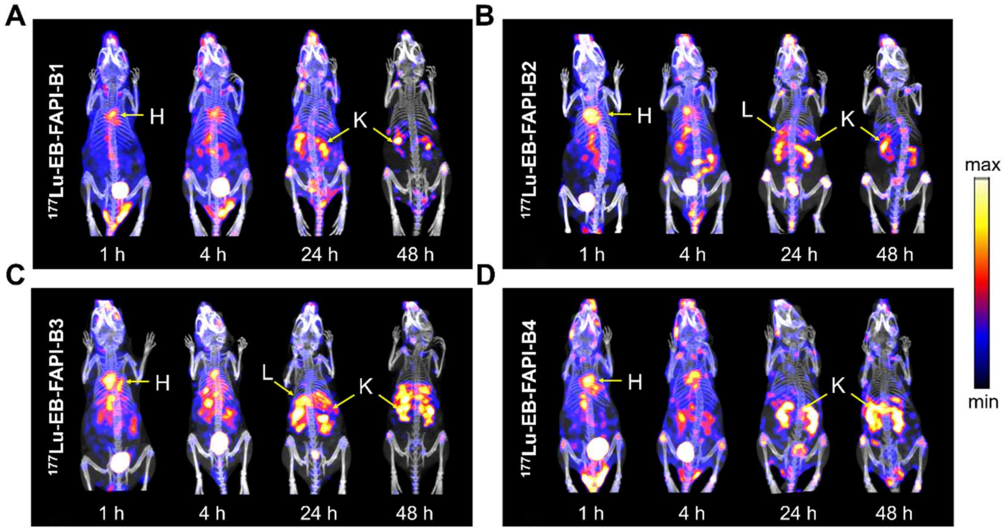

Figure S17. SPECT/CT imaging of ^{177}Lu-EB-FAPI-B1 **(A)**, B2 **(B)**, B3 **(C)**, B4 **(D)** in healthy ICR mice at 1, 4, 24 and 48 h p.i.

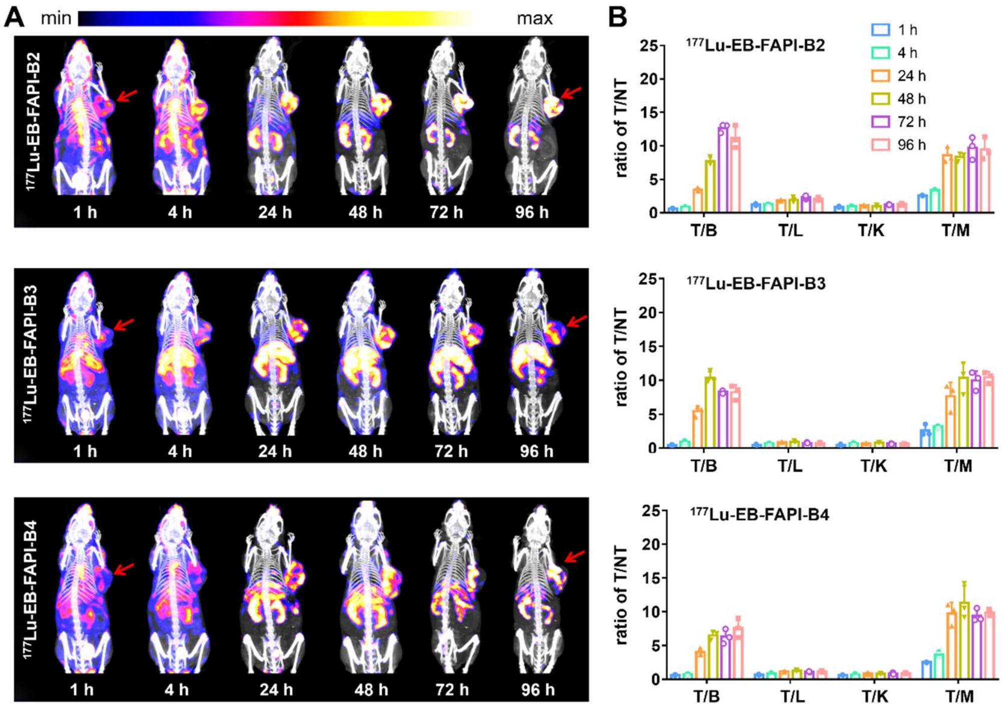

Figure S18. (A) SPECT/CT imaging of ^{177}Lu-EB-FAPI-B2, B3, B4 in U87MG tumor bearing mice at 1, 4, 24, 48, 72 and 96 h p.i. **(B)** The ratios of T/NT at different time points of SPECT imaging.

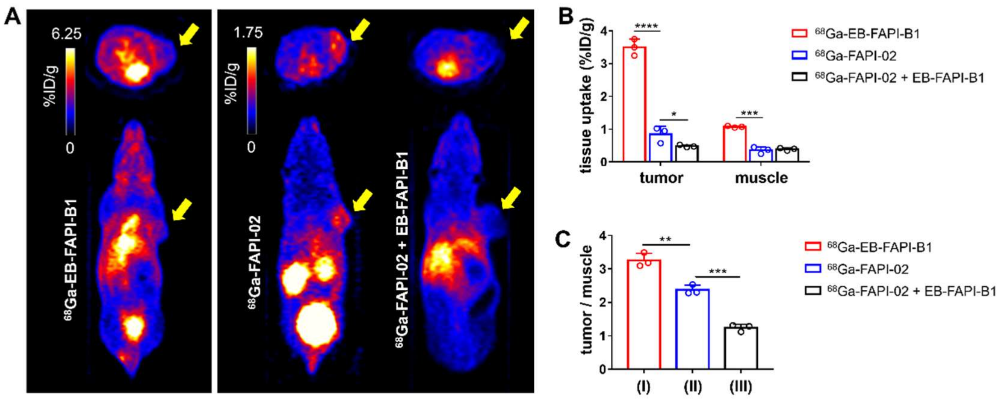

Figure S19. (A) PET imaging of ^{177}Lu-EB-FAPI-B1 in U87MG tumor-bearing mice at 1 h p.i. **(B)** The tissue uptake values of ^{68}Ga-EB-FAPI-B1, ^{68}Ga-FAPI-02 and blocking by EB-FAPI-B1 in the tumor and muscle. **(C)** The ratios of tumor/muscle after PET imaging of the radioligands.

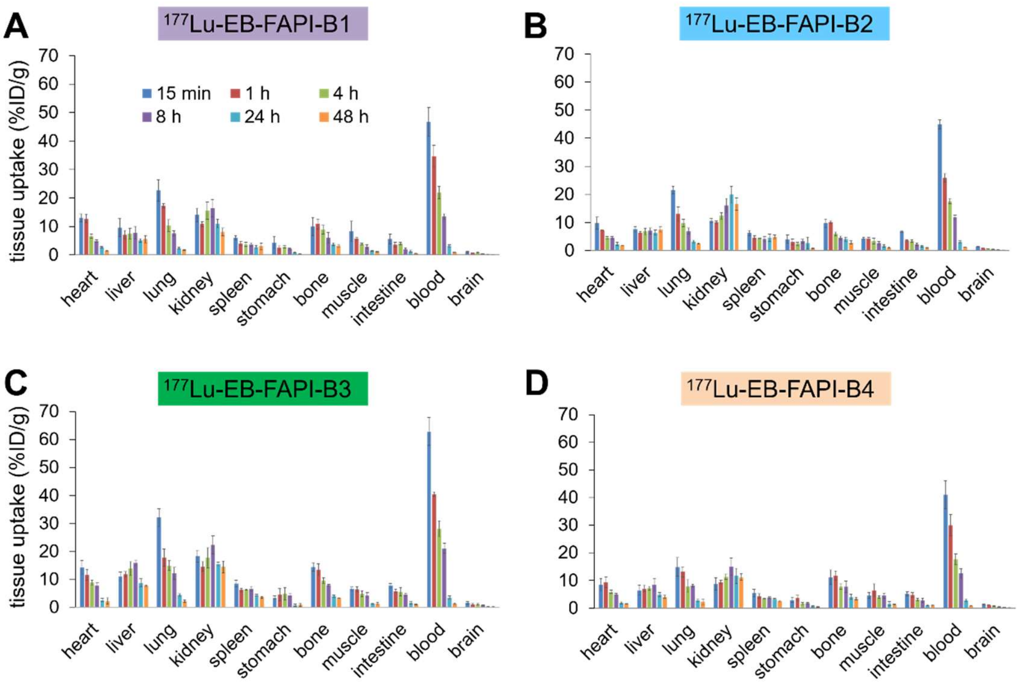

Figure S20. Biodistribution studies of ^{177}Lu-EB-FAPI-B1 **(A)**, B2 **(B)**, B3 **(C)** and B4 **(D)** in healthy ICR mice at 15 min, 1, 4, 8, 24 and 48 h after injection (n = 4/group).

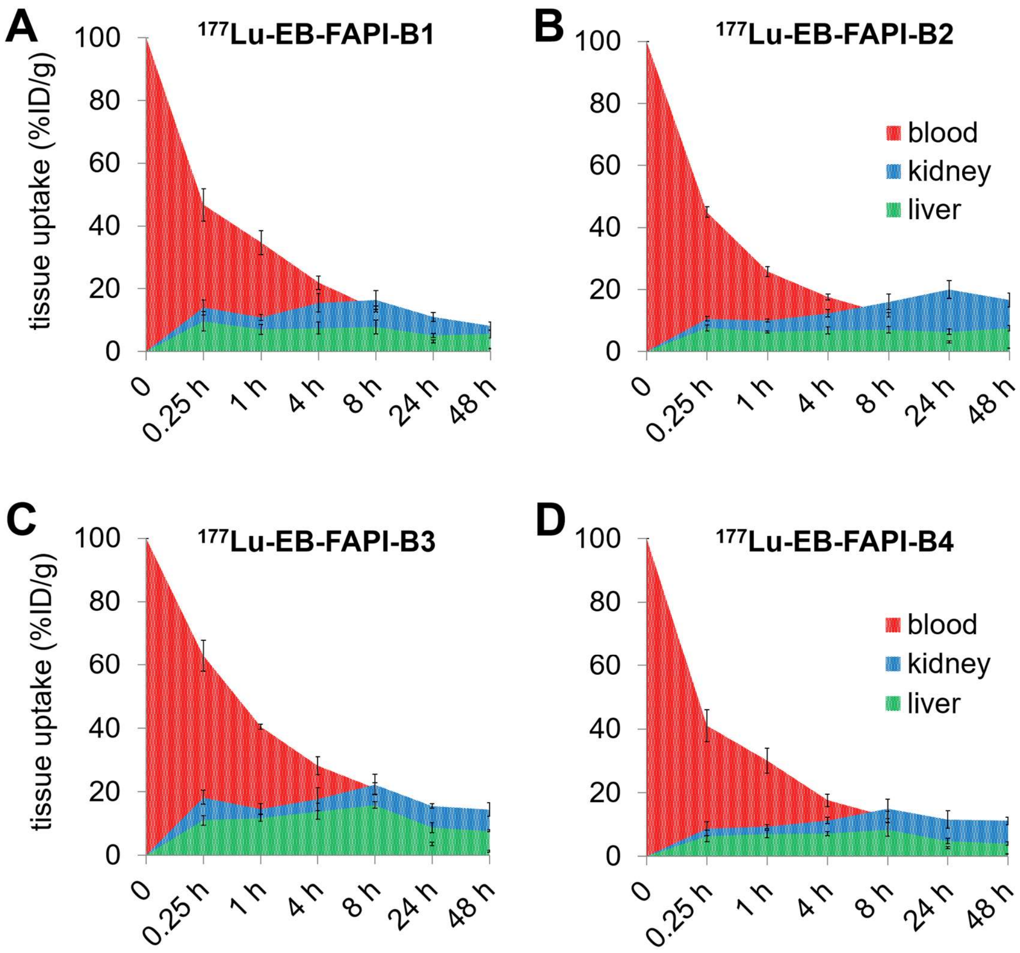

Figure S21. Tissue uptakes and variation trends of ^{177}Lu-EB-FAPI-B1 **(A)**, B2 **(B)**, B3 **(C)**, B4 **(D)** in the blood, kidneys, and liver at different times after injection.

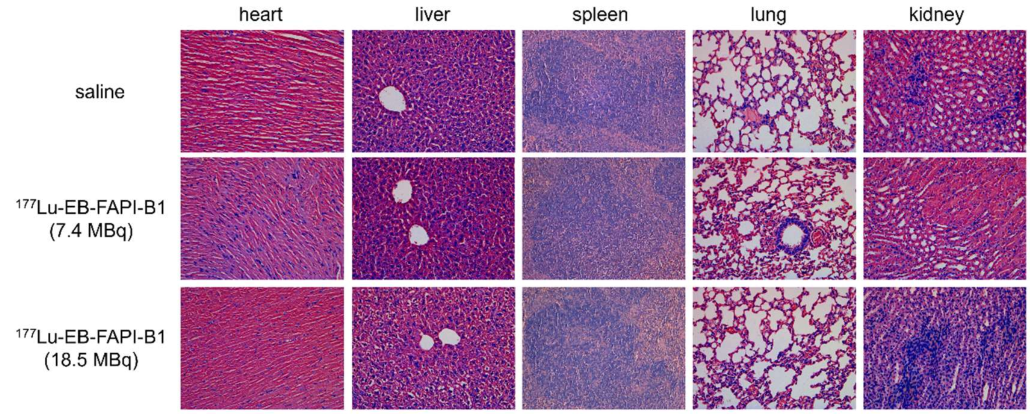

Figure S22. H&E staining of the major organs including heart, liver, spleen, lung and kidneys after treatment with saline, 7.4 MBq and 18.5 MBq of ^{177}Lu-EB-FAPI-B1, respectively.

Table S1. Radiochemical characteristics of the compounds.

**Compounds**	**^{177}Lu-EB-FAPI-B1**	**^{177}Lu-EB-FAPI-B2**	**^{177}Lu-EB-FAPI-B3**	**^{177}Lu-EB-FAPI-B4**
pH	5.0 - 5.4	5.0 - 5.4	5.0 - 5.4	5.0 - 5.4
Characteristics	Bluish violet and transparent	Bluish violet and transparent	Bluish violet and transparent	Bluish violet and transparent
Precursor (μg)	50	50	50	50
Radioactivity concentration (MBq/mL)	9.25-370	9.25-370	9.25-370	9.25-370
Specific radioactivity (MBq/nmol)	5.85-11.7	6.20-12.40	6.55-13.10	6.85-13.70
Radiochemical purity (%)	> 97	> 97	> 97	> 97
Radiolabeling yield (%)	> 97	> 97	> 97	> 97
Log P	-1.90 ± 0.12	-2.10 ± 0.04	-2.13 ± 0.11	-2.18 ± 0.004
